# *Chrysanthemum Morifolium–*Derived Nanovesicles Reduce Lactate‐Associated Histone Lactylation and Modulate Microglia‐Mediated Neuroinflammation in Sepsis‐Associated Encephalopathy

**DOI:** 10.1002/advs.78330

**Published:** 2026-10-14

**Authors:** Kaiqiang Li, Xuling Luo, Xiaoting Zhang, Chengwen He, Wei Xu, Aibo Xu, Shushan Hua, Liping Wang, Liang Ma, Ke Hao, Youmin Ying, Zhen Wang

**Affiliations:** ^1^ Center for Laboratory Medicine Allergy center Department of Transfusion medicine Zhejiang Provincial People's Hospital (Affiliated People's Hospital) Hangzhou Medical College Hangzhou Zhejiang China; ^2^ Key Laboratory of Biomarkers and In Vitro Diagnosis Translation of Zhejiang Province Hangzhou Zhejiang China; ^3^ College of Pharmaceutical Sciences Zhejiang University of Technology Hangzhou Zhejiang China; ^4^ Department of Pharmacy Taizhou Municipal Hospital (Taizhou University Affiliated Municipal Hospital) School of Medicine Taizhou University Taizhou Zhejiang China; ^5^ State Key Laboratory of Fluid Power and Mechatronic Systems School of Mechanical Engineering Zhejiang University Hangzhou Zhejiang China

**Keywords:** chrysanthemum morifolium, H3K18la/NF‐κB axis, hippocampus, microglial polarization, nerve injury, plant exosome‐like nanovesicles

## Abstract

Sepsis‐associated encephalopathy (SAE)—a complication of the central nervous system in patients with sepsis—markedly diminishes cognitive functions and is linked to hippocampal neuritis–induced injury. Plant exosome‐like nanovesicles can transport proteins, lipids, and molecular compounds between neural cells and the periphery, thereby modulating target cells. This study examined the therapeutic potential of *Chrysanthemum morifolium*–derived exosome‐like nanovesicles (CRELNVs) in an animal model of lipopolysaccharide‐induced SAE, revealing their ability to effectively cross the blood–brain barrier, be internalized by microglia, and alleviate nerve injury and memory impairment by reducing lactate levels in microglia at the hippocampus. CRELNVs decreased lactylation levels in microglia, thus promoting the conversion of microglial cells from the detrimental M1 metabolic phenotype to the protective M2 one. Mechanistically, our finding suggest CRELNVs reduced proinflammatory cytokine release is associated with the inhibition of NF‐κB activation. Thus, CRELNVs were concluded to exert their protective effects by modulating the H3K18la/NF‐κB axis and regulate microglia‐mediated neuroinflammation by responding to the hippocampal neuritis–induced injury in SAE.

## Graphical abstract

When used to treat sepsis‐associated encephalopathy, Chrysanthemum morifolium–derived nanovesicles can inhibit the H3K18la/NF‐κB axis to regulate microglia‐mediated neuroinflammation.

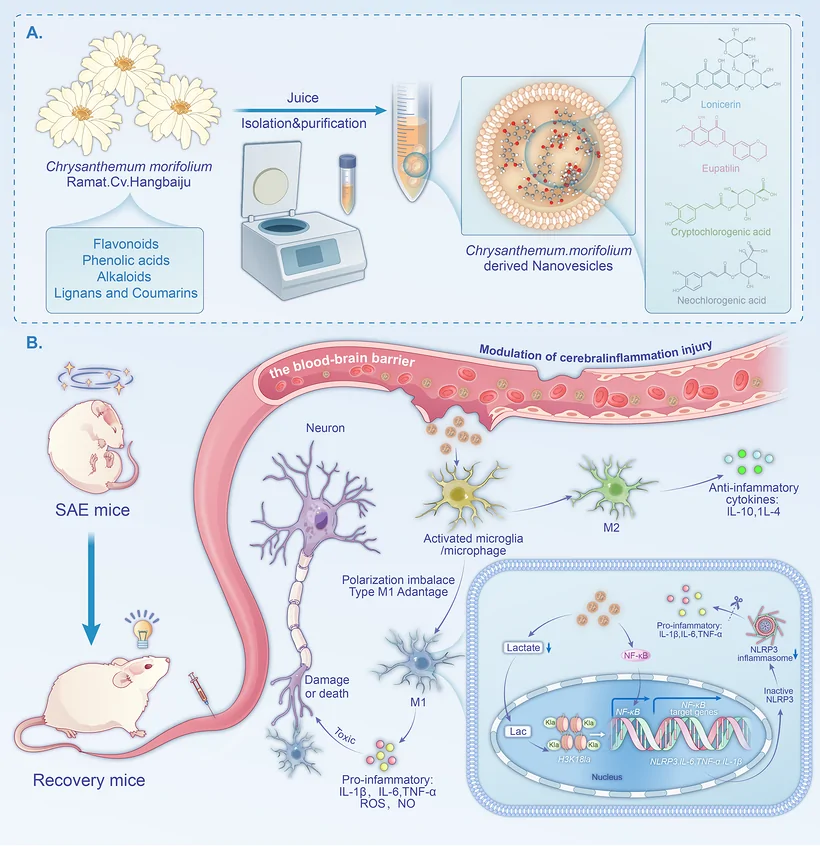

## 1 Introduction

Sepsis is an organ dysfunction that is caused by bacterial, fungal, viral, and parasitic infections and accounts for nearly 20% of global mortality [1]. Sepsis‐associated encephalopathy (SAE)—a central nervous system complication in patients with sepsis—leads to brain dysfunction due to systemic infection [2]. Reactive microglia contribute to synaptic impairment, neuronal death, and neurogenesis inhibition and thus cause SAE [3, 4]. An endogenous lactate‐related metabolic regulatory mechanism operating in microglia under SAE conditions allows them to phagocytose, present antigens, and produce inflammatory cytokines [5, 6]. Lactate disrupts homeostasis, making microglia release proinflammatory cytokines such as tumor necrosis factor‐α (TNF‐α), interleukin‐6 (IL‐6), and interleukin‐1β (IL‐1β), which induce spatial learning and memory disorders in mice [7, 8]. Therefore, agents controlling microglial homeostasis and regulating microglia‐mediated neuroinflammation in SAE should be developed.

Plant exosome‐like nanovesicles (PELNVs) are cell‐derived vesicles formed via the budding of endosomal membranes [9]. The superior molecular transport capabilities and biocompatibility of PELNVs make them valuable active substances for transmitting information in humans, plants, animals, and microbes [10, 11]. As PELNVs are biogenetically plant‐derived vesicles, their unique components may reflect typical effects in parental cells, particularly within herbal medicine [12, 13]. The bioactive components contained in PELNVs can greatly facilitate the correction of abnormal immunity and recovery from cell injury [14, 15, 16]. Moreover, PELNVs exert neuroprotective effects (protection against brain injury), attenuate ischemia‐reperfusion‐induced damage to the blood–brain barrier [17], and inhibit neuronal apoptosis [18].

*Chrysanthemum morifolium*, recognized for its anti‐inflammatory and antioxidant activities, is traditionally used in China to alleviate headaches and eye discomfort [19, 20]. Various components of *C. morifolium*, including flavonoids, phenolic acids, terpenes, and steroids, are pharmacologically active, e.g., exhibit anti‐inflammatory and anti‐oxidant effects and have the capacity to prevent chronic diseases [21]. *C. morifolium* modulates inflammatory responses, nuclear factor kappa B (NF‐κB) signaling pathways, lipid anabolism, renin‐angiotensin‐aldosterone, and metabolic abnormalities [22]. However, the low bioavailability and insufficient selectivity of active herbal ingredients complicate their widespread use [23, 24], and the components of *C. morifolium* responsible for its anti‐SAE effect and their functions are unknown.

Herein, exosome‐like nanovesicles isolated from fresh *C. morifolium* Ramat. cv. Hangbaiju (CRELNVs) are shown to be capable of reprogramming the lesion microenvironment in the context of SAE. In vivo experiments indicate that CRELNVs reduce lactate levels, balance microglial homeostasis, and contribute to the improvement of spatial learning and memory in mice with SAE. In vitro experiments indicate that CRELNVs inhibit lactylation and control M2 gene expression in BV2 cells (as revealed by the detection of H3K18la marks in microglia). Additionally, CRELNVs can reduce proinflammatory cytokine release by inhibiting the NF‐κB activation. Our study contributes to the development of a novel SAE treatment platform that controls microglial homeostasis and holds considerable promise for clinical transformation.

## 2 Materials and Methods

### 2.1 Materials

Fresh *C. morifolium* Ramat. cv. Hangbaiju was purchased from a local agricultural market (Hangzhou, China). Lipopolysaccharide (LPS) extracted from *Escherichia coli* serotype O55:B5 (Cat. No. L2630) was procured from Sigma‐Aldrich (Dorset, UK), and interferon‐gamma (IFN‐γ; Cat. No. HY‐P7071) was acquired from MedChemExpress. Exosome‐specific lysis buffer (Cat. No. R601), protease inhibitors (Cat. No. HY‐P1005/1006), Cell Counting Kit‐8 (CCK‐8) assay kit (Cat. No. A311‐01), Coomassie Brilliant Blue rapid staining kit (Cat. No. P1300), reverse transcription reagents (Cat. No. R223), and SYBR Green Master Mix (Cat. No. Q711) were sourced from Nanjing Vazyme Biotech Company Co., Ltd. (Nanjing, China). TRIzol Reagent (Cat. No. 15596026) and the Bicinchoninic Acid (BCA) protein assay kit (Cat. No. 23225) were acquired from Thermo Fisher Scientific. Beyotime Biotechnology (Shanghai, China) provided the Dil dye (Cat. No. C1036), DiR dye (Cat. No. C1038), Hoechst 33342 stain (Cat. No. C1022), Actin‐Tracker Green‐488 probe (Cat. No. C1033), phosphate‐buffered saline (PBS) (Cat. No. C0221A), sodium dodecyl sulfate‐polyacrylamide gel electrophoresis (SDS‐PAGE) (Cat. No. P0690), Triton X‐100 (Cat. No. ST1723), 4',6‐diamidino‐2‐phenylindole (DAPI) (Cat. No. C1006), citrate buffer (Cat. No. P0081), Endogenous Peroxidase Blocking Buffer (Cat. No. P0100A), RIPA Lysis Buffer (Cat. No. P0013B), reactive oxygen species (ROS) assay kit (Cat. No. S0033) and dexamethasone (DEX) (Cat. No. ST1254). Dulbecco's Modified Eagle Medium (DMEM) (Cat. No. AW‐001‐T) and Fetal Bovine Serum (FBS) (Cat. No. AW‐F0601S) were procured from Anwei‐sci (Shanghai, China). SH‐SY5Y [SHSY‐5Y] Cell Complete Medium (Cat. No. CM‐0208) were procured from Procell system (Wuhan, China). IL‐1β (Cat. No. KE10003), IL‐6 (Cat. No. KE10007), TNF‐α (Cat. No. KE10002), and interleukin‐10 (IL‐10; Cat. No. KE1008) Enzyme‐linked immunosorbent assay (ELISA) kits were procured from Proteintech Group (Wuhan, China). Antibodies for arginase‐1 (Arg‐1; Cat. No. 93668), ionized calcium‐binding adapter molecule 1, (Iba‐1; Cat. No. 17198), Cluster of Differentiation 86 (CD86, Cat. No. 19589), Cluster of Differentiation 206 (CD206, Cat. No. 24595), NF‐κB p65 (Cat. No. 8242), and phospho‐NF‐κB p65 (Cat. No. 3033) were sourced from Cell Signaling Technology. The NOD‐like receptor family pyrin domain‐containing 3 (NLRP3) antibody (Cat. No. T55651) was procured from Abmart (Shanghai, China). The inducible Nitric Oxide Synthase (iNOS) antibody (Cat. No. ab15323) was sourced from Abcam. Pan‐Kla (Cat. No. PTM‐1401) and H3K18la (Cat. No. PTM‐1406) antibodies were purchased from PTM Bio (Hangzhou, China). Allophycocyanin(APC)‐CD206 (Cat. No. 141708), CD86 (Cat. No. 105001), and phycoerythrin‐cyanine 7 (PE‐Cy7)‐conjugated cluster of differentiation 11b (CD11b) (Cat. No. 101216) antibodies were procured from BioLegend. Horseradish peroxidase (HRP)‐conjugated goat anti‐rabbit immunoglobulin G (IgG) antibody (Cat. No. HA1001) was sourced from HuaAn Biotechnology (Hangzhou, China). The L‐lactate assay kit (Cat. No. A019‐2‐1) was supplied by Nanjing Jiancheng Bioengineering Institute (Nanjing, China).

### 2.2 Animals and Cell Lines

Specific pathogen‐free (SPF)‐grade healthy male C57BL/6 mice (eight weeks old, 20–22 g) were sourced from GemPharmatech Co., Ltd. (Jiangsu, China). The Animal Ethics Committee of Zhejiang Provincial People's Hospital reviewed and approved all animal experiments (Approval No. 20240202200516621695). The personnel conducting animal experiments had obtained the Zhejiang Laboratory Animal Technician Qualification Certificate (Certificate No. 202304330).

The BV‐2 murine microglial cell line (Research Resource Identifier (RRID): CVCL_0182) was procured from Shanghai Anwei Biotechnology Co., Ltd. (China). The RAW 264.7 murine macrophage cell line (RRID: CVCL_0493) was sourced from Wuhan Procell Life Science & Technology Co., Ltd. (China). The SH‐SY5Y human neuroblastoma cell line (RRID: CVCL_0019) and bEnd.3 mouse brain endothelial cell line (RRID: CVCL_0170) were obtained from the Cell Bank of the Type Culture Collection, Chinese Academy of Sciences (Shanghai, China).

### 2.3 CRELNV Isolation and Purification

Fresh *C. morifolium* Ramat. cv. Hangbaiju inflorescences were thoroughly rinsed with clean water and then homogenized in precooled PBS using a tissue homogenizer (Beyotime Biotechnology, China). The homogenate was filtered through a 300‐mesh sieve and sequentially centrifuged at 1000 *g* (30 min), 5000 *g* (30 min), and 15000 *g* (60 min) using a microcentrifuge (Sorvall Legend 17, Thermo Scientific, USA) to eliminate large contaminant particles, including cells, protoplasts, plant fibers, cellular debris, and large vesicles. The supernatant from the final centrifugation was additionally centrifuged at 120000 *g* (1.5 h) using an ultracentrifuge (Sorvall WX, Thermo Scientific, USA). For further purification, the dispersion obtained by resuspending the resulting pellet was layered onto a sucrose density gradient (8%, 15%, 30%, 45%, and 60% solutions in 0.02 M Tris(Hydroxymethyl)Aminomethane Hydrochloride (Tris‐HCl) and centrifuged at 150000 *g* for 2 h. The 15–30% fraction was collected, diluted with Tris‐HCl buffer (15 mL), and centrifuged at 5000 *g* for 30 min to remove sucrose. The pellet was resuspended in cold PBS, and the resulting dispersion of purified CRELNVs was sterilized by passage through a 0.22 µm filter and stored at −80°C for subsequent characterization.

### 2.4 Physical Characterization of CRELNVs

The morphology of purified CRELNVs was observed by transmission electron microscopy following negative staining with 3% phosphotungstic acid, and their zeta potential was assessed using dynamic light scattering (DLS) (Zetasizer Nano ZSE, Malvern Instrument, UK). Particle size, concentration, and vesicle ratio were measured using a Nanocoulter I instrument (Shenzhen, China). Fourier transform infrared spectroscopy (Thermo Scientific, USA) was used to characterize the chemical structures of CRELNVs.

### 2.5 Characterization of CRELNVs‐Associated Proteins

Proteins were isolated from CRELNVs using an exosome‐specific lysis buffer and quantified using a BCA assay kit. Subsequently, samples were subjected to electrophoresis on 10% SDS‐PAGE gel. After electrophoresis, the gel was carefully removed and stained with Coomassie Brilliant Blue fast staining solution with complete coverage and shaken at room temperature for 1 h to achieve sufficient staining. The stain was removed, and the gel was destained until the background became nearly clear and observed using a gel imaging system.

### 2.6 In Vivo Imaging Experiments

For in vivo tracking, CRELNVs were labeled with a near‐infrared dye (DiR) by incubation at 4°C for 1 h in the dark followed by ultracentrifugation at 120000 *g* and 4°C for 1 h. The pellet was resuspended in PBS to produce a dispersion of DiR‐labeled CRELNVs (DiR‐CRELNVs). To reduce imaging interference, the head and abdominal areas of C57BL/6 mice were depilated 24 h before in vivo imaging. DiR‐CRELNVs were administered in equivalent doses through intravenous injection.

CRELNV‐treated mice were imaged using a small animal imaging system (PerkinElmer) at different time points. At 24 h postinjection, the mice were sacrificed, and the organs were dissected for *ex vivo* imaging.

### 2.7 SAE Animal Model and In Vivo Anti‐SAE Experiments

An SAE model was established via the intravenous injection of LPS (10 mg/kg). Mice were randomly divided into four groups: Control, LPS, LPS + CRELNVs‐Low (4 mg/kg), and LPS + CRELNVs‐High (8 mg/kg). At 2 h postinjection, the LPS and CRELNVs‐L/‐H groups intravenously received an equal dose of PBS or CRELNVs, with subsequent doses administered every two days. Behavioral tests were conducted on days 6 and 7. Following the experiment, the mice were euthanized, and brain tissues were collected for analysis.

### 2.8 Behavioral Experiments

Mice were acclimatized for 30 min in the testing room prior to behavioral experiments, with behavioral performance subsequently recorded and analyzed using Tracking Master software (Labmaze V3.0, Zhongshi Technologyy, China).

In the open‐field test, each mouse was placed in a square arena (50 cm × 50 cm × 40 cm) and allowed to explore freely for 8 min, with a camera installed directly above to record movement trajectories. Ethanol (75%) was sprayed to clean the apparatus after each test to remove the odor and waste left by the last mouse. Locomotor and exploratory behaviors across experimental groups were evaluated by analyzing the total distance moved and time spent in the central zone.

In the novel‐object recognition test, each mouse initially explored the empty arena freely for 10 min. Subsequently, two identical objects were placed ∼10 cm from opposite walls, and the mouse was permitted to explore both objects for 10 min to assess inherent preferences for object location. Following a 1 h interval, a familiar object was substituted with a novel one of similar shape, and the mouse was given 5 min to explore both objects freely. Exploration was characterized by orienting the nose toward the object within a 2 cm range. Labmaze V3.0 software was used to analyze the time spent exploring each object, and a recognition index was subsequently calculated.

In the Y‐maze test, the apparatus consisted of three identical arms (40 cm × 10 cm) arranged at an angle of 120° from each other. Mice were placed at the end of one of the three labeled arms (A, B, or C), facing the central area, and permitted to explore all arms freely for 8 min. The sequence and frequency of arm entries were documented to calculate the spontaneous alternation rate, which assesses working memory performance.

### 2.9 Histological Examination and Terminal Deoxynucleotidyl Transferase Mediated dUTP Nick‐End Labeling (TUNEL) Assay

Mouse brain tissues underwent standard paraffin embedding and sectioning procedures. Pathological hippocampus damage was evaluated using hematoxylin and eosin (H&E) staining. Nissl staining was applied to assess neuronal integrity and activity. Neuronal apoptosis was detected using the TUNEL assay, which included sections deparaffinization and rehydration, treatment with proteinase K (20 µg/mL, 37°C, 15 min), and incubation with the labeling solution followed by treatment with streptavidin‐HRP. Signals were developed using 3,3’‐diaminobenzidine (DAB), and sections were counterstained with hematoxylin prior to microscopic examination.

### 2.10 3D Cell Reconstruction

Brain tissues were dehydrated using PBS solutions with varying sucrose concentrations and subsequently embedded in optimal cutting temperature (OCT) to prepare frozen sections. After permeabilization with 0.1% Triton X‐100 and blocking with a rapid immunofluorescence blocking solution, the sections were incubated with anti‐Iba‐1 antibody (1:200) at 4°C for 24 h and then with a 488‐conjugated secondary antibody at 20°C for 1 h. Nuclei were stained with DAPI. For imaging, *z*‐stack images were acquired at 1 µm intervals using a super‐resolution confocal microscope (Leica, Germany) equipped with a 63 × oil immersion objective. An excitation wavelength of 488 nm and emission wavelength of 519 nm were employed to visualize the Iba‐1 protein. Images of microglial branch nodes (1024 × 1024 pixel) and total branch length were reconstructed and quantitatively analyzed using the “Filaments” module of the Imaris 10.1.0 software.

### 2.11 Multiplex Immunofluorescence Histochemistry

Paraffin‐embedded brain sections underwent antigen retrieval in citrate buffer (pH 6.0) and peroxidase blocking with 3% H_2_O_2_. The sections were blocked and incubated with the first primary antibody at 4°C for 24 h and then incubated with HRP‐conjugated secondary antibodies for 1 h at room temperature. Tyramide signal amplification (TSA) was performed for 10 min at room temperature in the dark using a 1:500 dilution of the TSA enhancer and tyramide‐fluorophore conjugate to visualize the initial target. Antibody complexes were eliminated using heat‐induced epitope retrieval in antigen retrieval buffer (95°C, 30 min). The staining cycle, including primary antibody application, HRP‐conjugated secondary antibody application, and TSA development, was sequentially repeated for the second and third targets. Finally, sections were mounted with DAPI‐containing antifade medium for fluorescence microscopy analysis.

### 2.12 Transcriptome Sequencing

The brain tissues were rapidly cryopreserved utilizing liquid nitrogen. Subsequent ribonucleic acid (RNA) extraction was conducted employing the TRIzol Reagent following tissue homogenization. RNA concentration and purity were quantified using a NanoDrop spectrophotometer, while RNA integrity was evaluated using the Bioanalyzer system. Samples that satisfied the established quality criteria proceeded to library construction. Polyadenylated mRNA enrichment was achieved using oligo(dT) magnetic beads followed by fragmentation with magnesium‐based reagents. First‐strand complementary DNA (cDNA) synthesis from the fragmented RNA was accomplished using reverse transcriptase. The synthesis of the second‐strand cDNA was facilitated by *E. coli* DNA polymerase I and RNase H, with deoxyuridine triphosphate (dUTP) incorporated during the process. The resulting double‐stranded cDNA underwent end repair, adenylation at 3' ends, and adapter ligation. Magnetic beads were used to select library fragments. The Uracil‐DNA glycosylase (UDG) enzyme digested the second strand containing dUTP. Final libraries with an average insert size of 300 ± 50 bp were generated through polymerase chain reaction (PCR) amplification. Gene sequencing was performed using a platform with a paired‐end 150 bp (PE150) configuration.

### 2.13 In Vivo Biocompatibility

To assess the biocompatibility of CRELNVs, mouse body weight was monitored daily for 6 days. The CRELNV group received an intravenous injection of CRELNVs (8 mg/kg) every two days, while the PBS group was administered an equal volume of PBS via the same route. Following this treatment, serum biochemical parameters related to liver (aspartate aminotransferase, AST; alanine aminotransferase, ALT) and renal (urea; creatinine, Cr) functions were measured using a fully automated biochemistry analyzer. The major organs (heart, liver, spleen, lung, and kidney) were collected for H&E staining.

### 2.14 Hemolysis Experiments

Mouse whole blood was centrifuged at 4°C and 3000 rpm for 15 min to obtain red blood cells, which were then incubated with ddH_2_O (Positive control (PC)), PBS (Negative control (NC)) and various concentrations of CRELNVs (100, 200, 400 and 800 µg/mL CRELNVs) at 37°C for 4 h. After centrifugation, supernatant optical density (OD) was recorded at 540 nm. Red blood cells incubated with PBS and deionized water served as negative and positive controls, respectively.

$$\begin{array}{ccc} \mathrm{Hemolysis}\;\mathrm{rate}\;\left(\%\right) & = & 100\%\;\times \;\left(OD_{\mathrm{sample}}\;-\;OD_{\mathrm{negative}}\right)\\ & & /\left(OD_{\mathrm{positive}}\;-\;OD_{\mathrm{negative}}\right) \end{array}$$

### 2.15 Cell Culturing

An in vitro neuroinflammation model was established using BV2 microglial cells. Various neuroinflammation‐related cell lines, including RAW264.7, bEnd.3, and SH‐SY5Y, were selected for parallel experiments to verify biosafety. BV2 and RAW264.7 cells were maintained in high‐glucose DMEM supplemented with 10% FBS. bEnd.3 cells were cultured in high‐glucose DMEM supplemented with 10% FBS and 1% penicillin–streptomycin. SH‐SY5Y cells were cultured in a SH‐SY5Y [SHSY‐5Y] Cell Complete Medium. All cells were maintained at 37°C in a humidified atmosphere containing 5% CO_2_ and passaged upon reaching 90% confluence.

### 2.16 Cellular Uptake Evaluation

CRELNVs were labeled with an appropriate amount of Dil (excitation wavelength: 549 nm; emission wavelength: 565 nm) as described in Section 2.6. BV2 cells were exposed to 200 µg/mL Dil‐CRELNVs for varying durations (0, 1, 4, 8, 12, or 24 h). A flow cytometer (Agilent, USA) with a phycoerythrin (PE) channel was used to evaluate cellular uptake efficiency at each interval, and confocal laser scanning microscopy (Leica, Germany) was used to capture fluorescence images following Actin‐Tracker Green‐488 and Hoechst 33342 staining.

### 2.17 Cytotoxicity Evaluation

Cell viability was assessed using the CCK‐8 assay. Cells (5 × 10^3^ per well) were cultured in 96‐well plates and exposed to CRELNVs at concentrations of 0–400 µg/mL for 24 h. Subsequently, the CCK‐8 solution (10 µL) was added, and the cells were incubated for 2 h. Absorbance at 450 nm was measured using a Biotek microplate reader.

### 2.18 Reverse Transcription Quantitative Polymerase Chain Reaction (RT‐qPCR)

BV2 microglial cells were seeded in six‐well plates (5 × 10^5^ cells per well), and treated with CRELNVs, LPS (100 ng/mL), IFN‐γ (20 ng/mL), dexamethasone (10 µM), or a control blank medium after adherence. Following 24 h incubation, the cells were collected, and total RNA was extracted using TRIzol Reagent and reverse‐transcribed into cDNA. Gene expression was quantified by RT‐qPCR (Applied Biosystems 7500, USA). All PCR primers were commercially synthesized by Sunya Biotech (Hangzhou, China).

### 2.19 Flow Cytometry

Cells were treated as described in Section 2.18. After digestion and harvesting, anti‐CD11b, anti‐CD86, and anti‐CD206 antibodies were used to stain the cells. The levels of CD86 and CD206 proteins in BV2 cells were then assessed using flow cytometry (Agilent).

### 2.20 Immunofluorescence

Cells cultured on polylysine‐coated coverslips in six‐well plates were fixed after treatment with 4% paraformaldehyde for 15 min, permeabilized with 0.1% Triton X‐100, blocked, incubated with anti‐iNOS and anti‐Arg‐1 antibodies overnight at 4°C, and then stained with fluorescent secondary antibodies and Hoechst 33342. After washing with PBS, the coverslips were carefully detached using a curved syringe needle, and residual liquid was removed by filter paper from the edges. Specimens were imaged using confocal laser scanning microscopy after being mounted with antifade medium. And iNOS and Arg‐1 were visualized at 590 nm (an excitation wavelength) and 617 nm (emission wavelength).

### 2.21 Intracellular Lactate Quantification

Cells were treated as described in Section 2.18. The cell pellet collected after this treatment was subjected to intracellular lactate quantification using the nitrotetrazolium blue chloride reduction method according to manufacturer's instructions.

### 2.22 Inflammatory Cytokine Quantification

Cells were treated as described in Section 2.18. Following treatment, the supernatant from each group was centrifuged at 5000 *g* for 5 min at 4°C to eliminate particulate matter and then processed according to kit manufacturer's instructions to assess the expression of inflammatory cytokines. The concentration of the target factor was determined using a standard curve based on absorbance at 450 nm. For animal tissue samples, hippocampal homogenates were prepared and analyzed following the procedure used for cell cultures.

### 2.23 Western Blot Assay

The expression levels of specific proteins in BV2 cells treated with lonicerin (40 µg/mL), eupatilin (10 µg/mL), cryptochlorogenic acid (20 µg/mL), neochlorogenic acid (10 µg/mL), mixture (lonicerin (33.68 µg/mL), eupatilin (0.072 µg/mL), cryptochlorogenic acid (20.80 µg/mL) and neochlorogenic acid (9.92 µg/mL) and CRELNVs (400 µg/mL) were analyzed by western blotting. Cells were collected and lysed with RIPA Lysis buffer to extract total protein following treatment. Quantitative analysis of proteins in each sample was performed by SDS‐PAGE electrophoresis followed by transfer to polyvinylidene fluoride (PVDF) membranes. The membranes were blocked at room temperature with 5% skim milk for 2 h, incubated overnight with specific primary antibodies at 4°C, and then incubated with an HRP‐conjugated secondary antibody. Protein bands were visualized using a chemiluminescence detection system and imaged with a gel imaging system (Bio‐Rad, USA). Animal tissue samples were processed using a similar procedure.

### 2.24 ROS Quantification

The antioxidant capacity of CRELNVs was evaluated using a ROS assay kit. 2’,7’‐Dichlorodihydrofluorescein diacetate (DCFH‐DA) was diluted at final concentration of 10 µM in serum‐free medium. The culture medium was removed, and the cells were incubated with the DCFH‐DA working solution (1 mL per well) at 37°C for 20 min and then washed with PBS three times. Intracellular fluorescence was examined using a laser scanning confocal microscope. Cells were also collected and quantitatively analyzed via flow cytometry.

### 2.25 Liquid Chromatography‐Tandem Mass Spectrometry (LC‐MS/MS)

The bioactive components of CRELNVs were analyzed using LC‐MS/MS‐based metabolomics. After freeze‐drying, the samples were extracted with 80% aqueous methanol containing internal standards. Three rapid freezing–thawing cycles were performed, followed by centrifugation to collect the supernatant, which was filtered through a 0.22 µm membrane. Chromatographic separation was carried out using an Agilent SB‐C18 column (1.8 µm, 2.1 mm × 100 mm) with ultrapure water and acetonitrile as mobile phases. The injection volume equaled 2 µL, and gradient elution at a flow rate of 0.35 mL/min and column temperature of 40°C was used. Specific multiple reaction monitoring (MRM) ion pairs were monitored for each eluted metabolite throughout the analytical time window. Metabolite identification was performed based on secondary spectral matching against the Maiwei Metabolite Database.

Lonicerin, eupatilin, cryptochlorogenic acid, and neochlorogenic acid in CRELNVs were qualitatively analyzed by LC‐MS/MS at Wuhan Pronets Testing Technology Co., Ltd. (Wuhan, Hubei). A liquid sample (0.5 mL) was placed in a 15 mL centrifuge tube (sample amount = 4.45 mg) and freeze‐dried. Then, 80% aqueous methanol (1.0 mL) was added and ultrasonicated for 30 min at room temperature. After that, the mixture were centrifuged at 5000 rpm for 5 min at 4°C. The supernatant was collected, filtered through a membrane, appropriately diluted, and immediately analyzed by LC‐MS/MS. Chromatographic separation was performed on an Agilent Poroshell 120 SB‐C18 column (30°C) using an injection volume of 5 µL and a flow rate of 0.4 mL/min. The mobile phase consisted of 0.1% formic acid in water (solvent A) and acetonitrile (solvent B), and the following gradient was applied. Standard stock solution was diluted with methanol and prepared a series concentration of standard solution (0.1, 0.5, 1.0, 5.0, 10, 50, 100, 500, and 1000 ng/mL). Mass spectrometric detection was performed in cation mode using MRM. Quantification was calculated by a triple quadrupole mass spectrometer (AB SCIEX QTRAP 5500, USA).

### 2.26 Metabolomic and Bioinformatic Analyses

The Traditional Chinese Medicine System Pharmacology (TCMSP, https://tcmspw.com/tcmsp.php) database was used to identify active compounds in *C*. morifolium [25]. These compounds were initially screened based on a drug‐likeness threshold of ≥0.18, and the metabolomic components of the obtained CRELNVs were compared with those of *C. morifolium* to identify common species.

The protein targets of related compounds were supplied by the SwissTargetPrediction Similarity Ensemble Approach and TCMSP database. “Sepsis” and “microglia” were used as keywords, and related targets were collected from the GeneCards database. CRELNVs–compounds–target and CRELNVs–compounds–target–disease networks were analyzed using Cytoscape 3.10.1 [26].

### 2.27 Molecular Docking

The efficacy of binding between the active compounds in CRELNVs and core target was analyzed using molecular docking. Ligand structure data file (SDF) structures were obtained from the PubChem database, and receptor 3D structures were sourced from the Structural Bioinformatics Protein Data Bank (https://www.rcsb.org/). The ligands were further processed by adding hydrogen atoms and assigning charges. Receptor proteins underwent dehydration, ligand removal, and docking site definition. Docking simulations were conducted using AutoDock Tools version 1.5.7. A docking energy of less than −5 kcal/mol generally signifies binding affinity between the target and compound of interest and was therefore used as a threshold. PyMOL Version 2.0 was used to visualize docking results, facilitating a thorough analysis of interactions between CRELNV components and core targets.

### 2.28 Molecular Dynamics (MD) Simulations

The amino acid sequence of the receptor protein (NF‐κB) was retrieved from Uniprot (www.uniprot.org) using the accession number Q04206. Chemical structures were obtained from the PubChem database (pubchem.ncbi.nlm.nih.gov). The molecular docking model was generated by Boltz‐2, and the binding affinity (expressed in kcal/mol) and docking poses were recorded [27, 28].

MD simulations were performed using a complex ligand/NF‐κB model and Assisted Model Building with Energy Refinement 24 [29]. Initial energy minimization was carried out using the steepest descent algorithm and conjugate gradient. Subsequently, NVT (constant number, volume, and temperature) and NPT (constant number, pressure, and temperature) equilibrium calculations were performed for the system ensemble simulation during a 100 ns process using a system temperature of 298.15 K, a system pressure of 1 atm, a collision frequency of 2 ps^−1^, and an integration step size of 2 fs. Trajectories were saved every 10 ps for subsequent analysis. Root mean square deviation (RMSD), root mean square fluctuation (RMSF), solvent‐accessible surface area (SASA), and radius of gyration (*R*_g_) and perform hydrogen bond analysis calculated by Molecular Mechanics/Generalized Born Surface Area (MM/GBSA) [30].

### 2.29 Statistical Analysis

GraphPad Prism version 9.5 was used for data analysis and graph generation. Results were expressed as means ± standard deviations. Data normality was assessed using the Shapiro‐Wilk test. Statistical significance analysis of normally distributed data were conducted by the *t*‐test and one‐way analysis of variance (ANOVA).

## 3 Results and Discussion

### 3.1 Physicochemical Properties of CRELNVs

CRELNVs were prepared through differential centrifugation, ultracentrifugation, and sucrose density gradient purification (Figure 1A), with the majority distributed in the 15–30% range (Figure 1B), and displayed the characteristic cup‐shaped structure of extracellular vesicles and a relatively uniform size (Figure 1C). Zeta potential is a key indicator for assessing the stability of nanosystems, with nanoparticles exhibiting a zeta potential between −30 and 30 mV considered stable [31]. The zeta potential of CRELNVs (−19.50 ± 1.10 mV) was inside this range (Figure 1D). Most nanoparticles exhibited sizes of 50–100 nm (average = 68 nm), with the polydispersity index equaling 0.28 and particle concentration equaling 9.97 × 10^11^ particles/mL (Figure 1E). After treatment with Triton X‐100, the average particle size decreased to 61 nm, the concentration decreased to 2.89 × 10^10^ particles/mL, and the calculated vesicle proportion was 97.10% (Figure 1F). The concentration of proteins in CRELNVs was 5.62 ± 1.39 mg/mL. Most proteins in CRELNVs had molecular weights of 28, 40, and 55 kDa (Figure 1G). The infrared spectra of the CR leaching solution and CRELNVs were nearly identical (Figure 1H), the main components including flavonoids, phenolic acids, alkaloids, lignans, coumarins, and terpenoids (Figure 1I), which demonstrated that the compounds in CRELNVs were well preserved.

**FIGURE 1:**
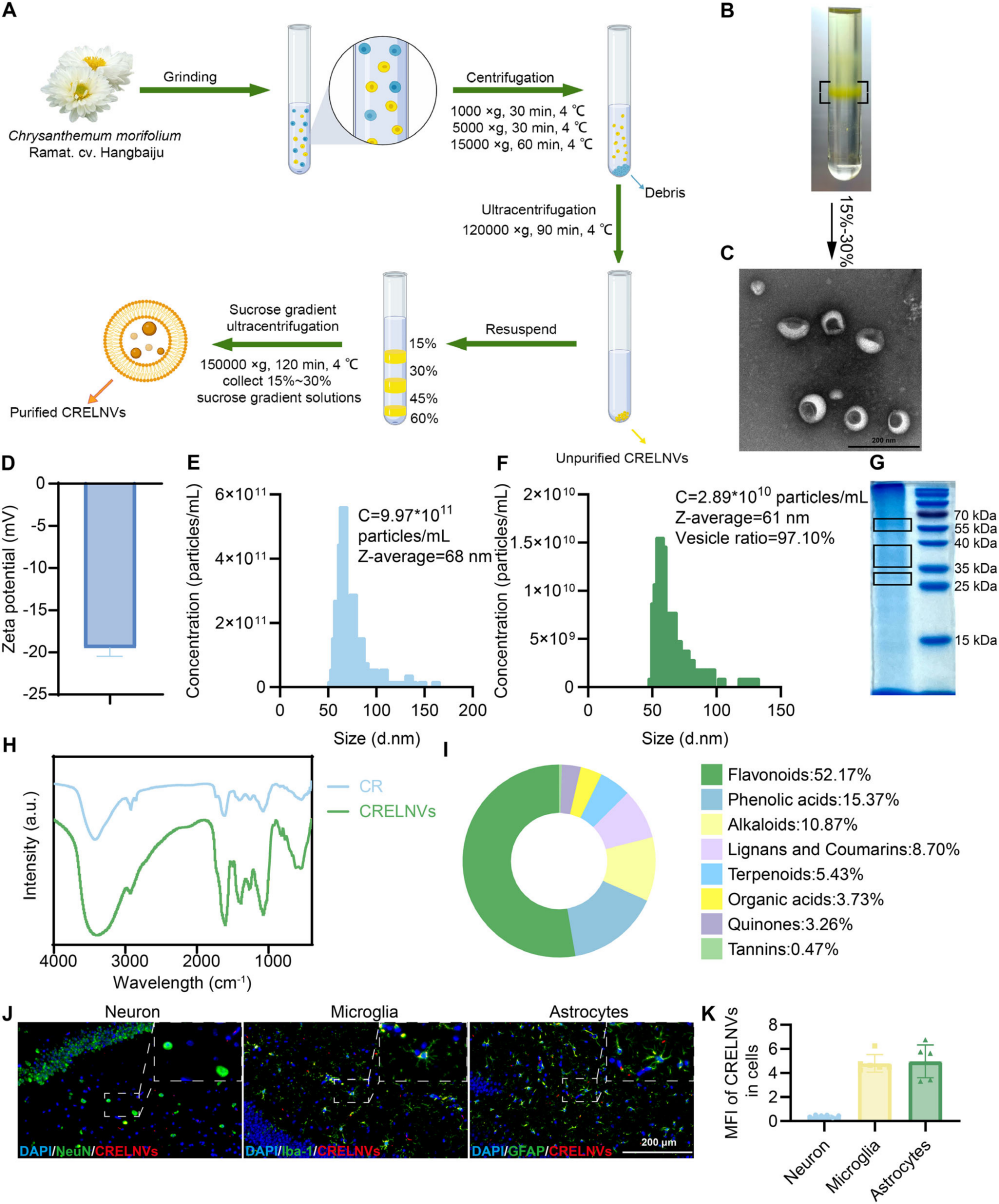
Preparation and characterization of CRELNVs. (A) Schematic of *Chrysanthemum morifolium*–derived exosome‐like nanovesicles (CRELNVs) preparation. (B) Size distribution of CRELNVs collected form sucrose gradient interfaces following ultracentrifugation. (C) Transmission electron microscopy image of CRELNVs. (D) Zeta potential of CRELNVs. Size and concentration of CRELNVs obtained (E) with and (F) without Triton X‐100 treatment. (G) Protein staining image of CRELNVs. (H) Infrared spectra of CRELNVs and the *Chrysanthemum morifolium* extract. (I) Proportions of various compounds in CRELNVs. (J) Representative immunofluorescence staining images showing NeuN, Iba‐1, and GFAP in murine hippocampi. (K) Quantification of fluorescence intensity in murine hippocampi, *n* = 6.

Given the structure of the blood–brain barrier and swift metabolic clearance mechanism of the brain [32], we investigated the in vivo targeting and metabolic duration of intravenously administered CRELNVs. CRELNVs entered the brain within 1 h following administration and were gradually metabolized, with brain fluorescence peaking at 12 h postinjection. Hence, the brain, heart, liver, spleen, lungs, and kidneys were collected for tissue imaging at 12 h postinjection. The brain exhibited fluorescence, which suggested that DiR‐CRELNVs had penetrated the brain tissue (Figure S1). To further investigate the cellular uptake of CRELNVs after their entry into the brain, we labeled them with Dil and observed their colocalization with major brain cells using immunofluorescence imaging. Dil‐CRELNVs were taken up by hippocampus cells, which included microglia, astrocytes, and neurons (Figure 1J,K). Microglia featured the highest uptake, and CRELNVs were therefore concluded to be more susceptible to phagocytosis by the mononuclear phagocyte system.

### 3.2 CRELNV‐Alleviated Memory Impairment in the Hippocampus of SAE Mice

Given the importance of hippocampal neuron damage in SAE, we explored the impact of CRELNVs on hippocampal neuritis–induced injury. Figure 2A illustrates the experimental timeline with brain analysis conducted 24 h postoperation. The body weight of SAE mice increased during seven days after CRELNVs administration (Figure S2A). Locomotor activity and cognitive function were evaluated using open‐field, novel‐object recognition, and Y‐maze tests on the sixth day after surgery. No significant variation in total traveled distance was observed between different groups (Figure S2B,C). The duration of murine activity in the central zone (Figure 2B) and novel object recognition time (Figure 2C) in the LPS+CRELNVs‐H group notably exceeded those in the LPS group. Moreover, the LPS+CRELNVs‐H group showed a significant increase in the spontaneous alternation rate in the Y‐maze test (Figure 2D). These results indicate that CRELNVs can improve cognitive disorder in SAE mice.

**FIGURE 2:**
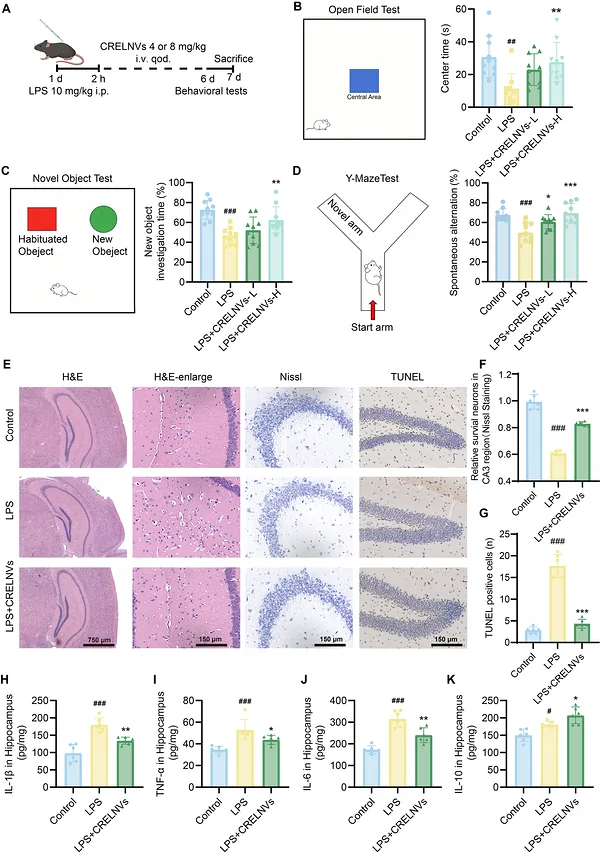
CRELNVs mitigated memory impairment in the hippocampus of SAE mice. (A) Schematic of the experimental procedure used to probe the effect of CRELNVs on memory impairment in the hippocampi of sepsis‐associated encephalopathy (SAE) mice. (B) Center times in open field test, *n* = 10. (C) Object discrimination times in novel‐object recognition test, *n* = 10. (D) Spontaneous alternation rates in Y‐Maze, *n* = 10. (E) Hematoxylin and eosin, Nissl, and TUNEL staining images. (F) Nissl staining quantitative analysis in CA3 region, n = 6. (G) TUNEL staining quantitative analysis in DG region, n = 6. (H) Interleukin‐1β (IL‐1β), (I) tumor necrosis factor‐α (TNF‐α), (J) interleukin‐6 (IL‐6), and (K) interleukin‐10 (IL‐10) levels in the hippocampi of SAE mice, *n* = 6. ^#^ *p* < 0.05, ^##^ *p* < 0.01, ^###^ *p* < 0.001 vs. control group; *p < 0.05, **p < 0.01, ***p < 0.001 vs. lipopolysaccharide (LPS) group.

To confirm the protective effect of CRELNVs, we explored their impact on hippocampal neurons in SAE. H&E staining revealed that CRELNV administration enhanced cellular alignment in the hippocampus in SAE mice (Figure 2E). Subsequently, the indistinctness or disappearance of Nissl bodies and the level of apoptosis were measured to characterize nerve cell damage. CRELNV treatment notably increased the number of Nissl bodies in the CA3 region (Figure 2F) and reduced that of apoptosis‐positive cells in the DG region (Figure 2G), as evidenced by Nissl and TUNEL staining. Additionally, cytokines in the hippocampal region were quantified using ELISA, with a significant reduction in the levels of proinflammatory cytokines (including IL‐1β, IL‐6, and TNF‐α) observed after CRELNV treatment (Figure 2H–K). Conversely, the level of the anti‐inflammatory cytokine IL‐10 significantly increased. These results suggest that CRELNVs may alleviate nerve injury in the hippocampal region by ameliorating neuroinflammation.

### 3.3 Effect of CRELNVs on Microglial Homeostasis in the Hippocampus of SAE Mice

The abnormal activation of the M1 microglial phenotype is an important cause of persistent neuroinflammation and exacerbated nerve injury [33]. We employed Iba1 to label microglia and examine the impact of CRELNVs on their quantity and morphology. The LPS group displayed an elevated number of microglia within the hippocampus (Figure 3A), whereas treatment with CRELNVs decreased this number (Figure 3B). The Imaris software was used for the 3D modeling of microglia to quantify branch length and the number of branch nodes. Treatment with CRELNVs significantly increased branch length and the number of branch nodes in the hippocampus of SAE mice (Figure 3C,D). RT‐qPCR was used to assess the metabolic phenotype of activated microglia, revealing that CRELNV treatment significantly decreased the mRNA expressions of IL‐1β, IL‐6, and TNF‐α and increased that of IL‐10 (Figure 3E–H). Additionally, CD86 and CD206 markers were employed to confirm the microglial phenotype in the hippocampus of SAE mice. CRELNVs significantly reduced CD86 expression and increased CD206 expression (Figure S3), which agreed with the trends in microglial homeostasis.

**FIGURE 3:**
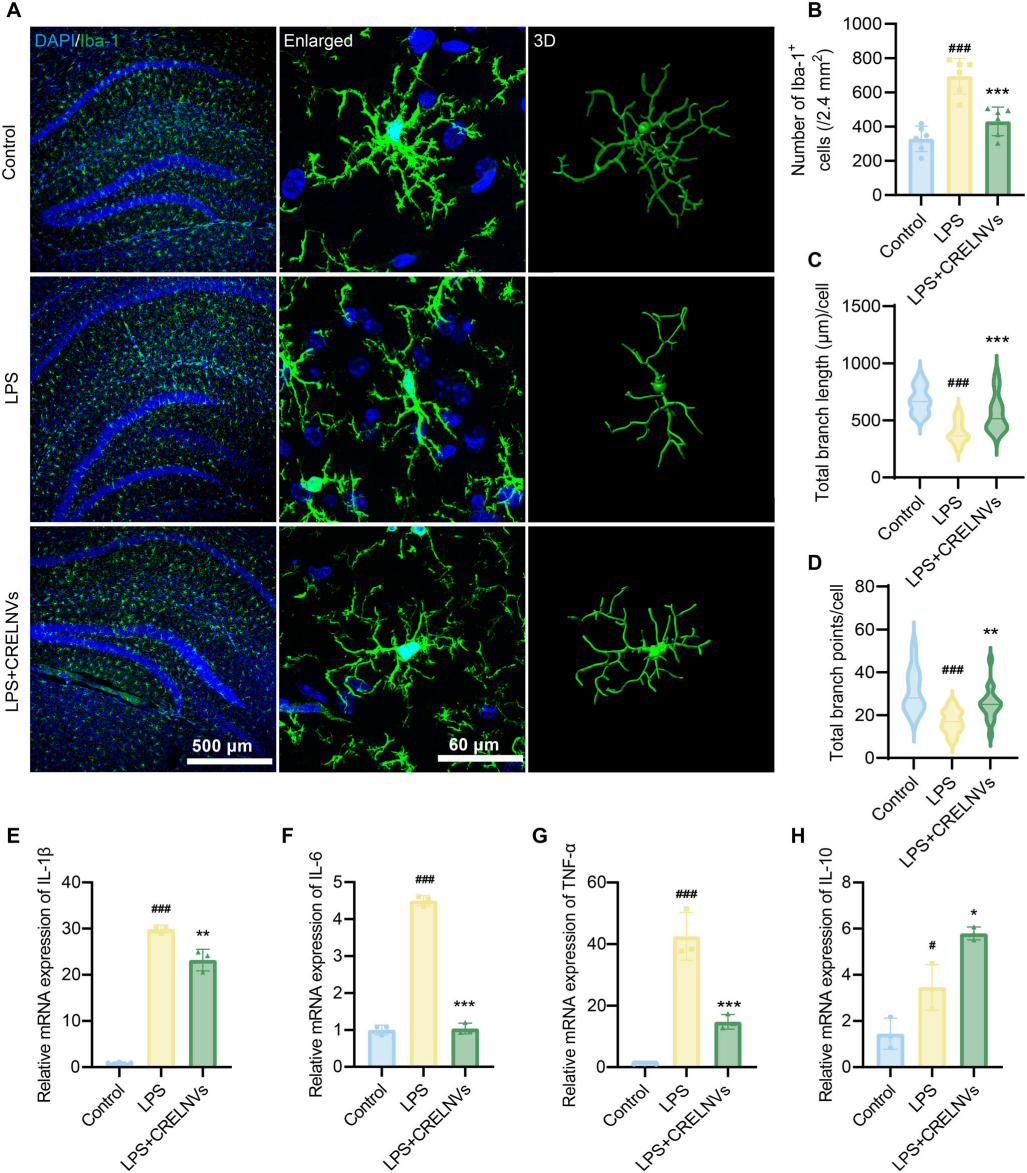
CRELNVs influence microglial homeostasis in the hippocampus of SAE mice. (A) Representative images of immunofluorescence staining for Iba‐1in the hippocampi of SAE mice. (B) Number of Iba‐1^+^ cells, (C) number of total branch points/cell, and (D) total branch length for panel A, *n* = 6. (E) IL‐1β, (F) TNF‐α, (G) IL‐6, and (H) IL‐10 mRNA expression in the hippocampi of SAE mice determined using the quantitative polymerase chain reaction (qPCR), *n* = 3. ^#^ *p* < 0.05, ^##^ *p* < 0.01, ^###^ *p* < 0.001 vs. control group; *p < 0.05, **p < 0.01, ***p < 0.001 vs. LPS group).

Lactate—a product of glycolysis in M1 microglia—is an important indicator of sepsis severity and mortality [34]. Microglia feature a lactate‐associated histone lactylation that can alter brain lactate levels through lactylation, which controls the metabolic phenotype of M1 cells [5]. We used ELISA and immunofluorescence assays to measure hippocampal lactylation levels. Compared with the LPS group, the LPS+CRELNVs group exhibited lower lactate concentrations and decreased lactylation levels in the hippocampus (Figure 4A–C). These results suggest that CRELNVs promote the transition of microglia from the detrimental M1 metabolic phenotype to the protective M2 phenotype by inhibiting lactate‐driven histone lactylation therein.

**FIGURE 4:**
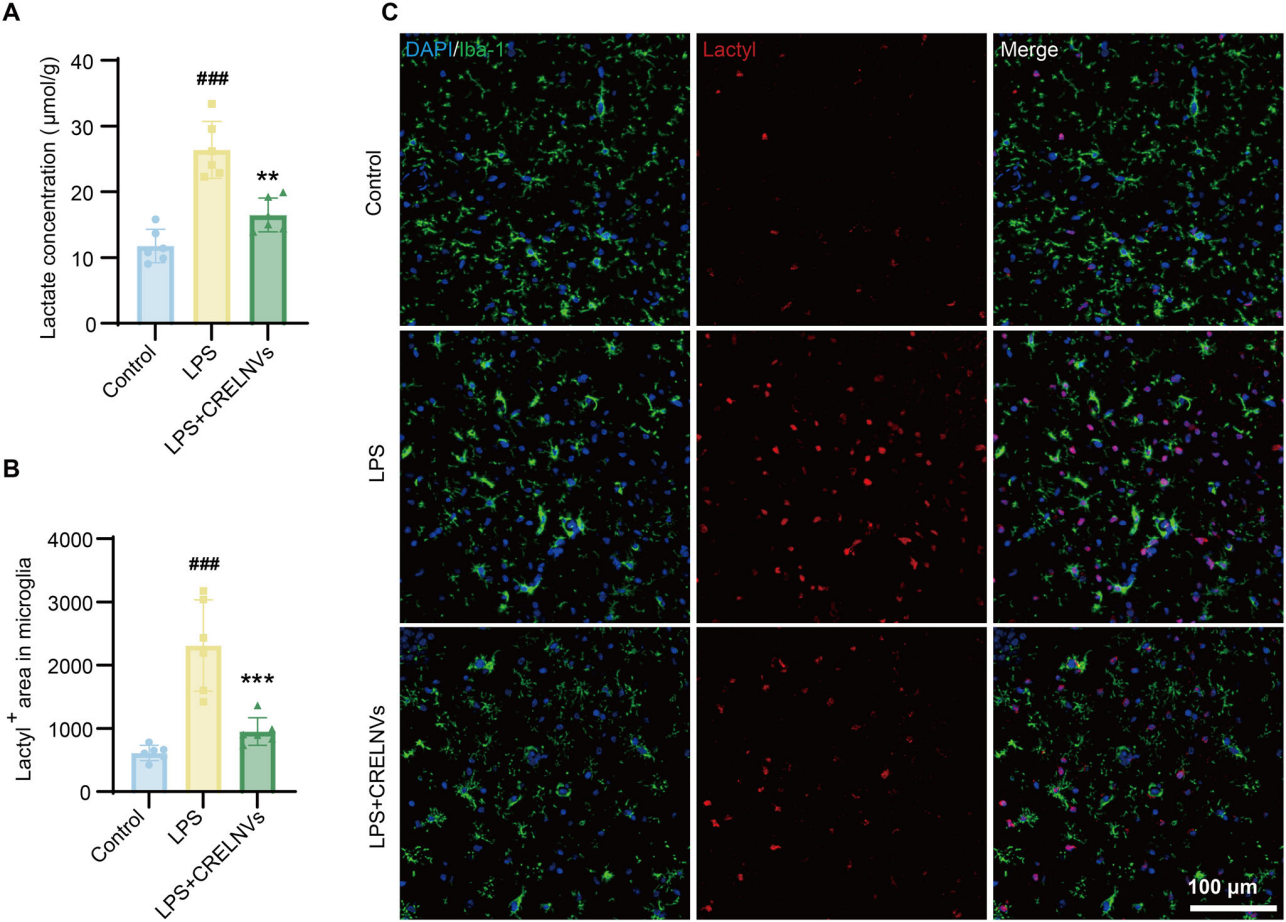
CRELNVs regulate the endogenous lactylation in the hippocampus of SAE. (A) Lactate concentration in hippocampi of SAE mice, *n* = 6. (B) Lactyl^+^/Iba‐1^+^ cell ratios obtained for panel C, *n* = 6. (C) Representative images of immunofluorescence staining for Iba‐1 and lactyl in the hippocampi of SAE mice. ^#^ *p* < 0.05, ^##^ *p* < 0.01, ^###^ *p* < 0.001 vs. control group; *p < 0.05, **p < 0.01, ***p < 0.001 vs. LPS group.

### 3.4 Regulatory Effect of CRELNVs on Inflammatory Responses Through NF‐κB in the Hippocampus of SAE Mice

We conducted transcriptome sequencing for the hippocampus of SAE mice to explore the mechanisms behind the therapeutic effects of CRELNVs on SAE. Principal component analysis revealed that LPS and LPS+CRELNVs groups were visible and clustered (Figure 5A). The statistics of differentially expressed genes (DEGs) and a volcano plot identified 962 DEGs. The LPS+CRELNVs group showed 343 upregulated and 619 downregulated genes compared with the LPS group (Figure 5B,C). Gene ontology (GO) term enrichment analysis indicated that mRNA targets were primarily associated with the immune system process, innate immune response, and inflammatory response (Figure 5D). Kyoto Encyclopedia of Genes and Genomes (KEGG) pathway analysis suggested that CRELNVs are potential modulators of microglial homeostasis processes related to nucleotide‐binding oligomerization domain (NOD)‐like receptor, PI3K‐Akt, TNF, and NF‐κB signaling pathway (Figure 5E). Gene set enrichment analysis identified significant enrichment in all GO terms and KEGG pathways, except for the PI3K‐Akt signaling pathway, which suggests that NF‐κB and NLRP3 play roles in determining the anti‐inflammatory efficacy of CRELNVs in the hippocampus of SAE mice (Figure 5F,G).

**FIGURE 5:**
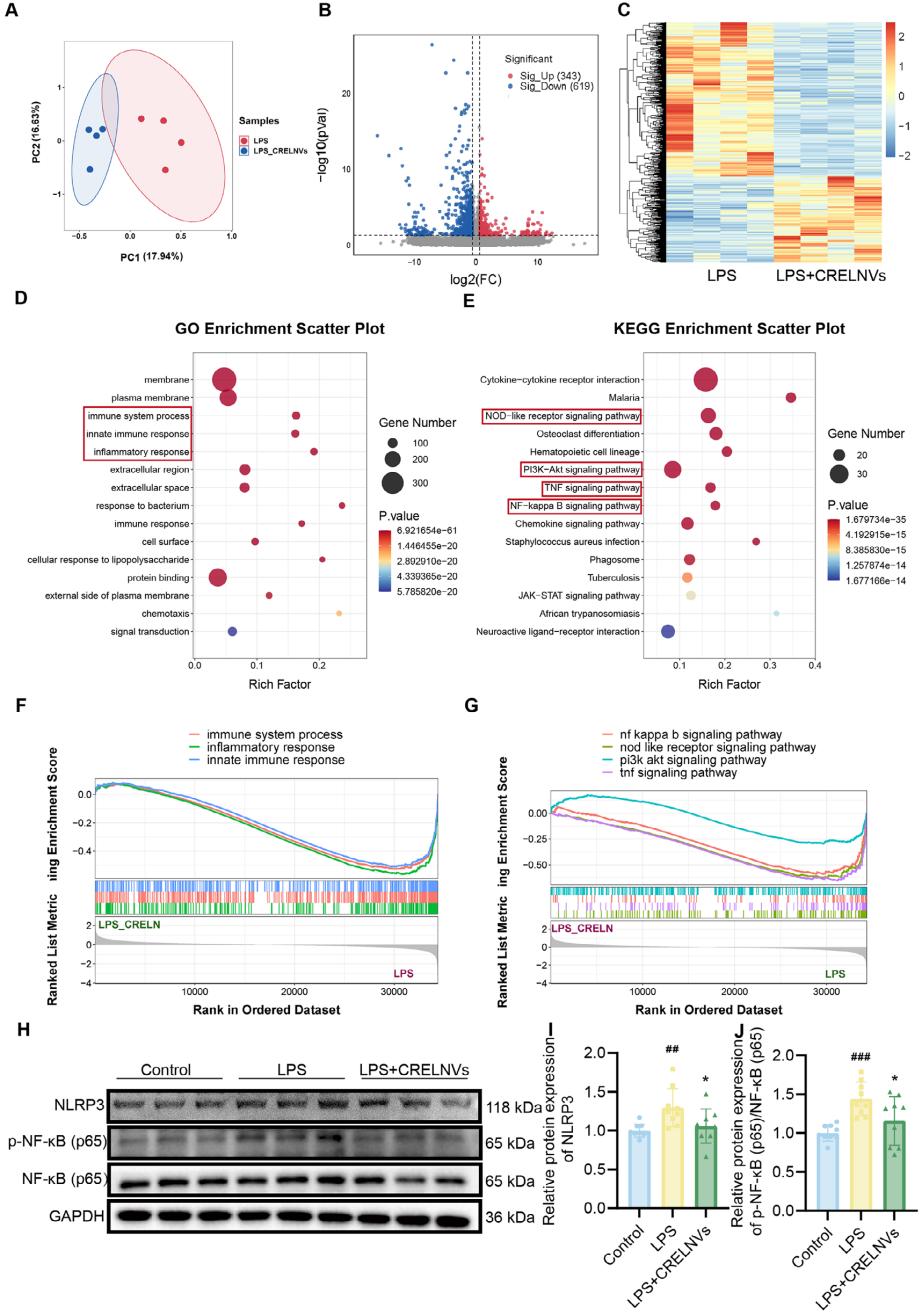
CRELNVs exert anti‐inflammatory effects via NF‐κB signal in the hippocampus of SAE mice. (A) Principal component analysis plot, (B) volcano plot of genomic analysis, and (C) heatmap showing that CRELNVs exert anti‐inflammatory effects via NF‐κB signaling in the hippocampi of SAE mice, *n* = 4. (D) GO enrichment analysis bubble graph of the top 15 signaling pathways, *n* = 4. (E) Enrichment analysis bubble graph of the top 15 signaling pathways, *n* = 4. (F) Gene ontology gene set enrichment analysis (GSEA) and (G) KEGG GSEA results, *n* = 4. (H–J) Western blot analysis results: NLRP3, p‐NF‐κB (p65), and NF‐κB (p65) levels in the hippocampi of SAE mice, *n* = 9. ^#^ *p* < 0.05, ^##^ *p* < 0.01, ^###^ *p* < 0.001 vs. control group;*p < 0.05, **p < 0.01, ***p < 0.001 vs. LPS group.

NF‐κB activation contributes to pro‐inflammatory signaling and NLRP3 inflammasome priming [35, 36]. Western blot analysis demonstrated that CRELNVs markedly reduced NF‐κB phosphorylation and decreased NLRP3 expression, indicating a reduction in inflammatory signaling (Figure 5H–J). Multiplex immunohistochemistry (mIHC) revealed that CRELNVs reduced NLRP3 expression in microglia (Figure S4). These findings support an association between CRELNVs treatment and attenuation of NF‐κB/NLRP3 inflammatory signaling.

### 3.5 In Vitro Neuroprotective Effect of CRELNVs

CRELNVs, which found be internalized by microglia and provoke anti‐inflammatory responses to protect nerve cells, exhibited no significant cytotoxicity in vivo (Figure S5). Specifically, no cytotoxicity was observed in BV2, RAW264.7, SH‐SY5Y, and bEnd.3 cells, and a time‐dependent uptake pattern was observed in BV2 cells in vitro (Figure S6). CRELNVs mitigated the reduction in SH‐SY5Y cell viability caused by the inflammatory active medium in a dose‐dependent manner (Figure 6A), thus having the capacity to alleviate experimental neurodamage without exhibiting cytotoxic effects.

**FIGURE 6:**
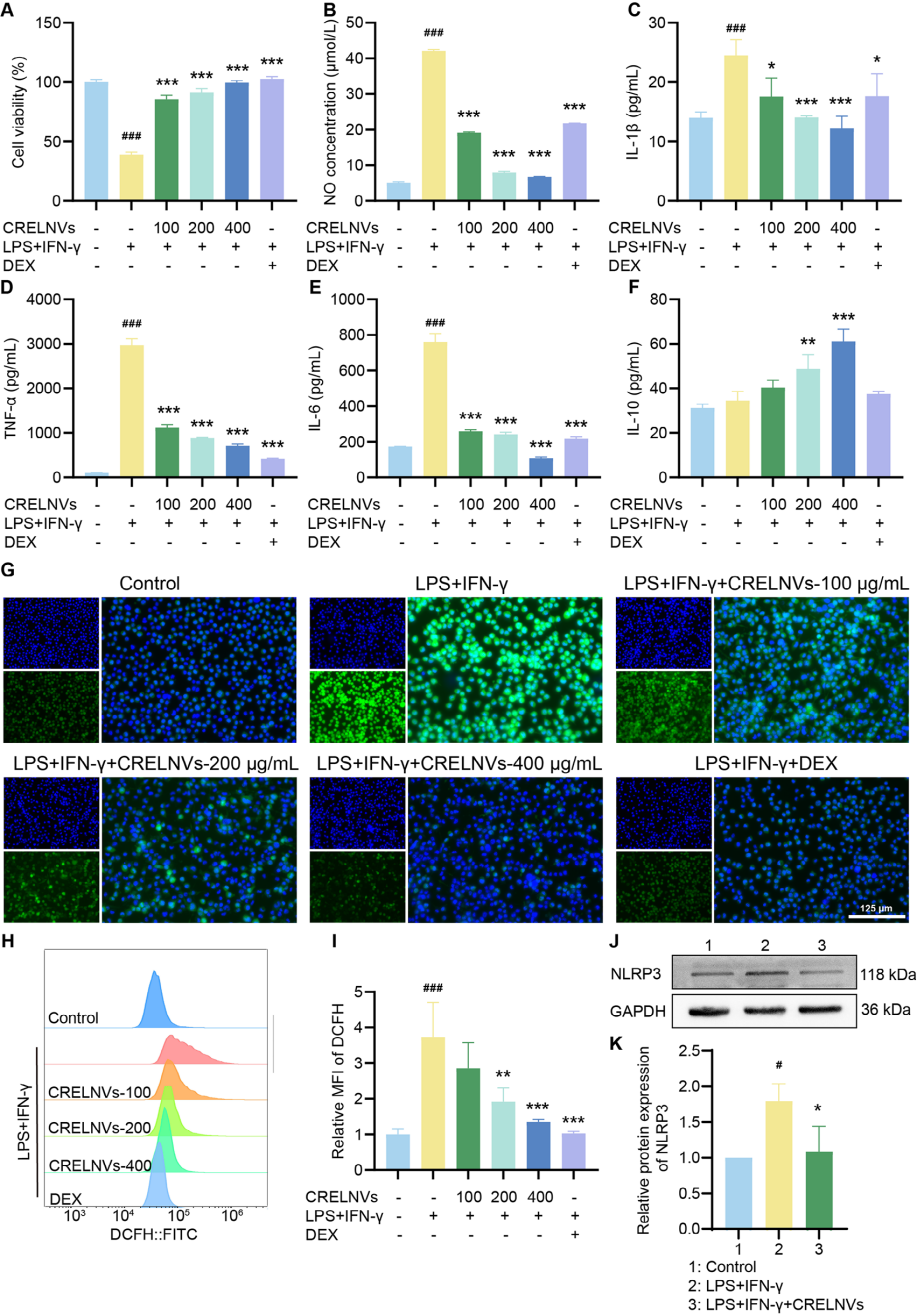
The neuro‐protection and Anti‐inflammation activities of CRELNVs in vitro. (A) Protective effect of CRELNVs on SH‐SY5Y cells in an inflammatory environment determined using the CCK8 assay, *n* = 4. (B) Microplate reader–detected NO contents in BV2 cell supernatants, *n* = 3. (C) IL‐1β, (D) IL‐6, (E) TNF‐α, and (F) IL‐10 levels in BV2 cell supernatants, *n* = 3. (G) Representative fluorescence microscopy images acquired to evaluate reactive oxygen species (ROS) content. (H, I) Flow cytometry peaks and ROS contents derived therefrom, *n* = 3. (J, K) Western blot analysis: NLRP3 levels in BV2 cells, *n* = 3. ^#^ *p* < 0.05, ^##^ *p* < 0.01, ^###^ *p* < 0.001 vs. control group; *p < 0.05, **p < 0.01, ***p < 0.001 vs. LPS group.

Microglia‐mediated NLRP3 inflammasome regulation plays an important role in neural inflammation injury [37]. In the priming phase of the NLRP3 inflammasome, LPS and endogenous factors such as TNF‐α and IL‐1β can trigger NLRP3 expression. Figure 6B–F demonstrate that CRELNVs reduced the levels of proinflammatory cytokines (such as NO, IL‐1β, TNF‐α, and IL‐6) while enhancing the secretion of the anti‐inflammatory cytokine IL‐10 in the LPS and IFN‐γ–induced inflammatory model. The extensive release of proinflammatory cytokines initiates an inflammatory cascade, increasing cellular ROS levels and activating the NLRP3 inflammasome [38]. CRELNVs dose‐dependently reduced ROS levels in the inflammatory model, exerting effects comparable with those of DEX at a concentration of 400 µg/mL (Figure 6G–I), and inhibited NLRP3 expression in BV2 cells (Figure 6J,K). These findings suggest that CRELNVs regulate inflammatory cytokine expression and reduce ROS levels and inhibit the NLRP3 inflammasome, contributing to nerve cell protection [39].

### 3.6 In Vitro Effects of CRELNVs on Lactylation and the Metabolic Phenotype of Microglia

To evaluate the phenotype of microglia, we used flow cytometry, focusing on CRELNVs associated with the expression of pro‐ and anti‐inflammatory cytokines. After CRELNV administration, the CD86‐positive rate significantly decreased, while the CD206‐positive rate markedly increased (Figure 7A–C). The anti‐inflammatory macrophages induced by metabolic reprogramming are primarily characterized by M1 phenotype downregulation [40], which results in decreased iNOS expression, and M2 phenotype upregulation [41], which results in elevated Arg‐1 expression. Using IHC and RT‐qPCR, we observed a significant decrease in iNOS expression and significant increase in Arg‐1 expression following CRELNV treatment (Figure 7D–I), which suggests that CRELNVs inhibit M1 polarization while promoting M2 polarization in vitro.

**FIGURE 7:**
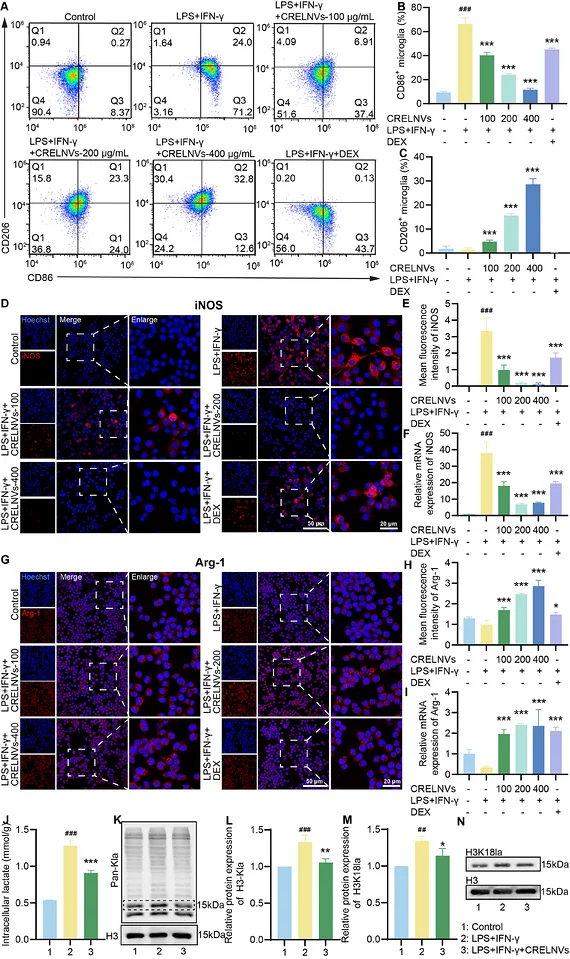
CRELNVs controlled lactylation and modulated the metabolic phenotype of microglia in vitro. (A) Representative flow cytometry quadrant plots for CD86^+^ and CD206^+^ cells. Fractions of (B) CD86^+^ and (C) CD206^+^ cells. (D) Representative images of immunofluorescence staining for iNOS in BV2 cells. (E) iNOS fluorescence intensity in BV2 cells, *n* = 3. (F) iNOS mRNA expression in BV2 cells quantified using the qPCR, *n* = 3. (G) Representative images of immunofluorescence staining for Arg‐1 in BV2 cells. (H) Quantification of Arg‐1 fluorescence intensity in BV2 cells, *n* = 3. (I) Arg‐1 mRNA expression in BV2 cells quantified using the qPCR, *n* = 3. (J) Lactate concentration in BV2 cells, *n* = 3. (K, L) Western blot analysis: Pan‐Kla and H3 levels in BV2 cells, *n* = 3. (M, N) Western blot analysis: H3K18la and H3 levels in BV2 cells, *n* = 3. ^#^ *p* < 0.05, ^##^ *p* < 0.01, ^###^ *p* < 0.001 vs. control group; *p < 0.05, **p < 0.01, ***p < 0.001 vs. LPS group.

Lactate is a vital end‐product of glycolysis and an important indicator of energy metabolism, altering the immune microenvironment of microglia [42]. CRELNV treatment mitigated the rise in lactate levels in BV2 cells induced by inflammatory modeling (Figure 7J). H3K18 lactylation serves as a biomarker for diagnosing and predicting septic shock [6, 43]. The Western Blot assay was used to detect protein panlactylation and H3K18 lactylation levels in BV2 cells. After LPS and IFN‐γ treatment, lactylation mainly occurred at H3 (15 kDa) and H4 (11 kDa) histones, and the lactylation levels at both sites decreased after CRELNVs treatment (Figure 7K,L). The H3K18la lactylation level also significantly decreased after CRELNVs treatment (Figure 7M,N and Figure S7). These results demonstrate that CRELNVs can downregulate lactate levels and inhibit histone lactylation (H3K18la) in a time‐dependent manner in microglial cells and thus modulate their metabolic phenotype [44].

### 3.7 In Vitro Effect of CRELNVs on Inflammatory Responses via NF‐κB Axis

To gain further insight into the molecular basis of NF‐κB inhibition observed in vivo (Figure 5H), we performed integrative metabolomic and network pharmacological analyses on CRELNVs. This approach was intended to identify potential bioactive constituents within CRELNVs that might contribute to the observed anti‐inflammatory activity, rather than to definitively establish causal relationships at this stage. Metabolomic analysis enabled the identification of 644 compounds in CRELNVs, including flavonoids (52.17%), phenolic acids (15.37%), alkaloids (10.87%), lignans/coumarins (8.70%), terpenoids (5.43%), organic acids (3.73%), quinones (3.26%), and tannins (0.47%) (Table S1). Figure S8A illustrates the 12 compounds common between the 84 active compounds identified in *C. morifolium* using the TCMSP database and the metabolic components of CRELNVs, with each common compound having a relative content exceeding 1 × 10^7^. And the relative content of these compounds in CRELNVs were shown in Figure S9A–C. Notably, 95 genes were common between the 342 target genes of CRELNVs and 1186 sepsis‐related genes (Figure S8B). The protein‐protein interaction (PPI) network revealed the involvement of the NF‐κB signaling pathway, which includes IL‐6, TNF, NF‐κB1, and CASP3 (Figure S8C). Molecular docking was repeated three times, and the results indicated that lonicerin, eupatilin, cryptochlorogenic acid, and neochlorogenic acid are closely associated with microglia‐mediated neuroinflammation in sepsis, including PTGS2, SIRT1 and NFKB1 (Figure S8D,E and Table S2), which have a relationship with NF‐κB involved immune responses [45, 46].

It was reported that flavonoids and phenolic acids were significantly involved with NF‐κB responses [47]. Furthermore, it can prevent behavioral deficits and dopaminergic neuron degeneration in a mouse model of neurologic damage disease [48]. Subsequently, the interactions between NF‐κB and the key active compounds of CRELNVs (lonicerin, eupatilin, cryptochlorogenic acid, and neochlorogenic acid) were explored using MD simulations [30]. First, we selected the vast space of possible binding region of NF‐κB protein surfaces with these 4 active compounds by Boltz‐2 method. As shown in Figure 8A and Figure S10A, Lonicerin interacts stably with NF‐κB within a superficial pocket, facilitated by hydrogen interactions. (SER‐401, SER‐377 and LEU‐379) and hydrophobic interaction (LEU‐403 and LEU‐379). Eupatilin interacts stably with NF‐κB within a superficial pocket, facilitated by Hydrogen interaction (ASN‐186 and ARG‐35) and Aromatic skeleton‐driven non‐covalent recognition (ARG‐33). Cryptochlorogenic‐acid and Neochlorogenic‐acid interact stably with NF‐κB within a superficial pocket, facilitated by Salt Bridge (LYS‐56 and ARG‐35, and ARG‐124, respectively), Hydrogen interaction (ASP‐53 and ARG‐33, and ALA‐461, LYS‐123 and ARG‐33, respectively) and hydrophobic interaction (PRO‐47 and LYS‐28, and THR‐464, respectively). Then, the MD simulations were used. The complex components exhibited an affinity of binding energies when docked with NF‐κB (Table S4), as analyzed by MM‐GBSA, and these binding energies were significantly lower than those from these single‐Molecule dynamics simulation of each active components (Table S5). A 100 ns molecular dynamics simulation confirmed the binding compatibility between the active components and NF‐κB, with convergence to low‐energy states (Figure 8B), sustained 2–20 hydrogen bonds (Figure 8C), and stable RMSD and Rg after 20 ns (Figure 8D,E). The complex of active components‐NF‐κB were structurally and energetically stable, as shown in the protein RMSF and complex SASA analysis (Figure 8F,G). And the ligand RMSD and total contributing residues of these 4 compounds in CRELNVs were shown in Figure S10B,C. Based on our predictions, the effect of CRELNVs on NF‐κB is likely mediated by the synergistic action of their components, including lonicerin, eupatilin, cryptochlorogenic acid, and neochlorogenic acid. However, because computational docking and MD simulations provide only predictive evidence, we next performed cellular thermal shift assays (CETSA) and Western blot analysis to experimentally verify whether CRELNVs directly affect NF‐κB signaling in BV2 microglial cells.

**FIGURE 8:**
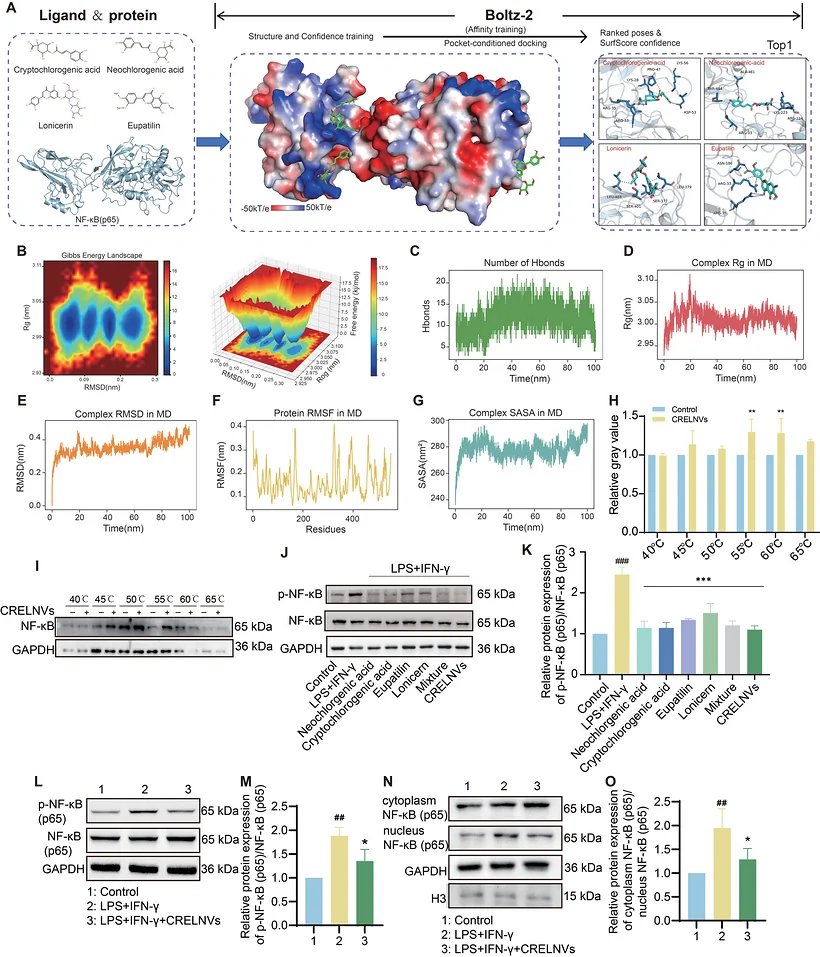
CRELNVs modulated NF‐κB activation. (A) Molecular docking pose of active components bound to NF‐κB. (B) Free‐energy landscape of the active components and NF‐κB complex from a 100 ns MD simulation; red and blue regions denote high‐ and low‐energy basins, respectively. (C) Time course of the number of hydrogen bonds in the complex. (D) Time course of the gyration radius of the complex. (E) Time course of the root mean square deviation of the complex. (F) Root mean square fluctuation of the complex. (G) Time course of the solvent‐accessible surface area of the complex. (H, I) CETSA, *n* = 3. (J–O) Western blot analysis: p‐NF‐κB p65 and NF‐κB p65 levels in BV2 cells, *n* = 3. ^#^ *p* < 0.05, ^##^ *p* < 0.01 vs. control group; *p < 0.05, **p < 0.01 vs. LPS group.

Furthermore, we analyzed the expression patterns of NF‐κB p65 and its phosphorylated form (p‐NF‐κB p65) in BV‐2 cells treated with CRELNVs to verify the inhibiting of NF‐κB activation. And the content of these 4 active compounds in CRELNVs were shown in Table S3 by LC‐MS/MS. Then, Cellular thermal shift assay (CETSA) confirmed that CRELNVs significantly enhanced the thermal stability of the NF‐κB protein (Figure 8H,I). And these 4 compounds and CRELNVs had a significant inhibition of p‐NF‐κB p65, respectively (Figure 8J–M). Additionally, Western blot analysis in Figure 8L–O indicated a significant activation of NF‐κB p65 in the model control group, evidenced by increased p‐NF‐κB p65/NF‐κB p65 and cytoplasmic NF‐κB p65/nuclear NF‐κB p65 ratios. In contrast, treatment with intact CRELNVs effectively reversed these changes, demonstrating that CRELNVs as a whole exert potent inhibition of NF‐κB activation [49]. Notably, our metabolomic and computational analyses identified several bioactive compounds within CRELNVs, including lonicerin, eupatulin, cryptochlorogenic acid, and neochlorogenic acid. While these individual constituents may participate in the observed molecular interplay with NF‐κB, the contribution of the complete nanovesicle cargo (rather than any single compound) appears to underpin the robust anti‐inflammatory efficacy.

## 4 Conclusions

This study highlights the potential of CRELNVs isolated from fresh *C. morifolium* Ramat. cv. Hangbaiju for SAE treatment. CRELNVs act as effective agents against nerve injury in the hippocampal region by regulating microglial homeostasis. Our findings demonstrate that CRELNVs effectively alleviate hippocampal nerve injury and cognitive deficits in SAE mice by reducing lactate‐associated histone lactylation and promoting microglial M2 polarization. Mechanistically, we provide both in vivo and in vitro evidence that CRELNVs inhibit neuroinflammation through suppression of the H3K18la/NF‐κB axis. Integrative metabolomic and computational analyses suggest that this effect may involve multiple bioactive constituents, including lonicerin, eupatilin, cryptochlorogenic acid, and neochlorogenic acid, acting in a synergistic manner. However, given the current lack of selective compound depletion data, the precise causal contribution of each individual component remains to be established in future studies employing targeted add‐back rescue approaches. In conclusion, we have developed a platform that exhibits remarkable therapeutic efficacy and responds to the brain microenvironment, thereby alleviating hippocampal neuritis–induced injury.

## Author Contributions

K.L. wrote original draft, performed methodology, data curation, and funding acquisition. X.L. performed methodology, data curation, and wrote the original draft. X.Z. performed methodology and data curation. C.H. performed formal analysis and investigation. W.X. performed formal analysis and investigation. A.X. performed methodology. S.H. performed visualization. L.W. performed data curation and validation. L.M. performed supervision, review and editing. K.H. performed supervision, wrote, review and edited the final manuscript. Y.Y. review and edited the final manuscript. Z.W. performed project administration, funding acquisition, wrote, review and edited the final manuscript.

## Declaration of generative AI and AI‐assisted technologies in the manuscript preparation process

Home for Researchers((https://www.home‐for‐researchers.com/))was used in the preparation of this manuscript for the purpose of reference collection. All references collected by this tool were manually reviewed and verified by the authors to ensure their accuracy, relevance, and appropriateness. The use of this AI tool did not influence the key arguments or conclusions of the manuscript, as all analyses and conclusions are based on the authors’ understanding and judgment of the primary sources. The authors takes full responsibility for the content and accuracy of this work.

## Conflicts of Interest

The authors declare no conflicts of interest.

## Supporting information




**Supporting File 1**: advs78330‐sup‐0001‐S1‐10.pdf.

**Supporting File 2**: advs78330‐sup‐0002‐S1.xlsx.

**Supporting File 3**: advs78330‐sup‐0003‐S2‐S6.docx.

## Data Availability

The data that support the findings of this study are available from the corresponding author upon reasonable request.
