# Supplementary material for: Chrysanthemum Morifolium–Derived Nanovesicles Reduce Lactate‐Associated Histone Lactylation and Modulate Microglia‐Mediated Neuroinflammation in Sepsis‐Associated Encephalopathy
Source: Adv Sci (Weinh). 2026 Oct 14:e78330. Online ahead of print. doi: 10.1002/advs.78330 (PMC13669446; doi:10.1002/advs.78330)
Supplement: Supplementary file 1 [file ADVS-9999-e78330-s002.pdf]

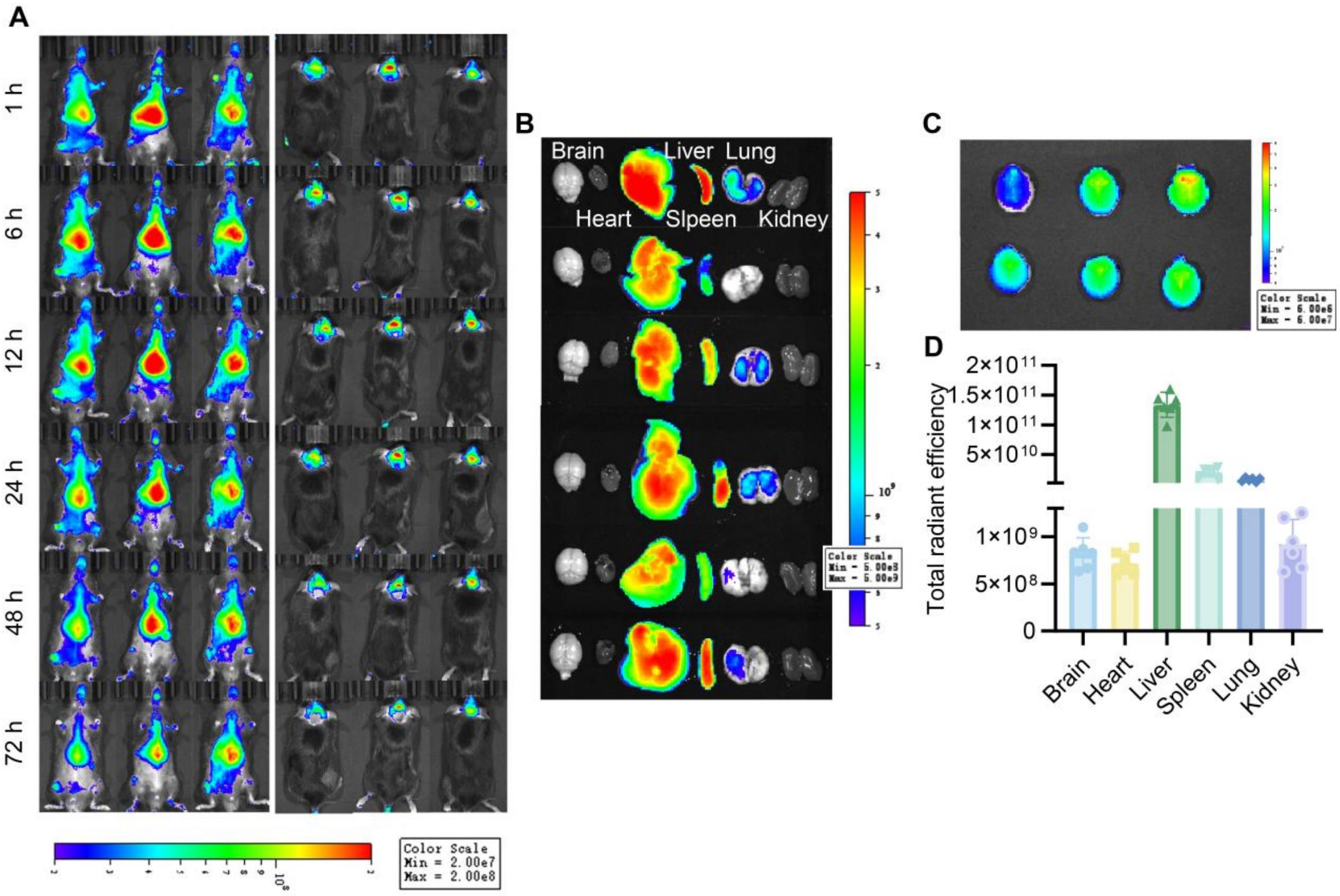

Figure S1 In vivo uptake of DiR-CRELNVs in mice. A. Representative IVIS images of mice at 1h, 6h, 12h, 24 h, 48h and 72h after injection of DiR-labeled CRELNVs, n=3. B-C. Representative IVIS images of organs harvested from mice at 12h after intravenous injection of DiR-CRELNVs. D. Quantification of the fluorescence intensity in organs, n=6.

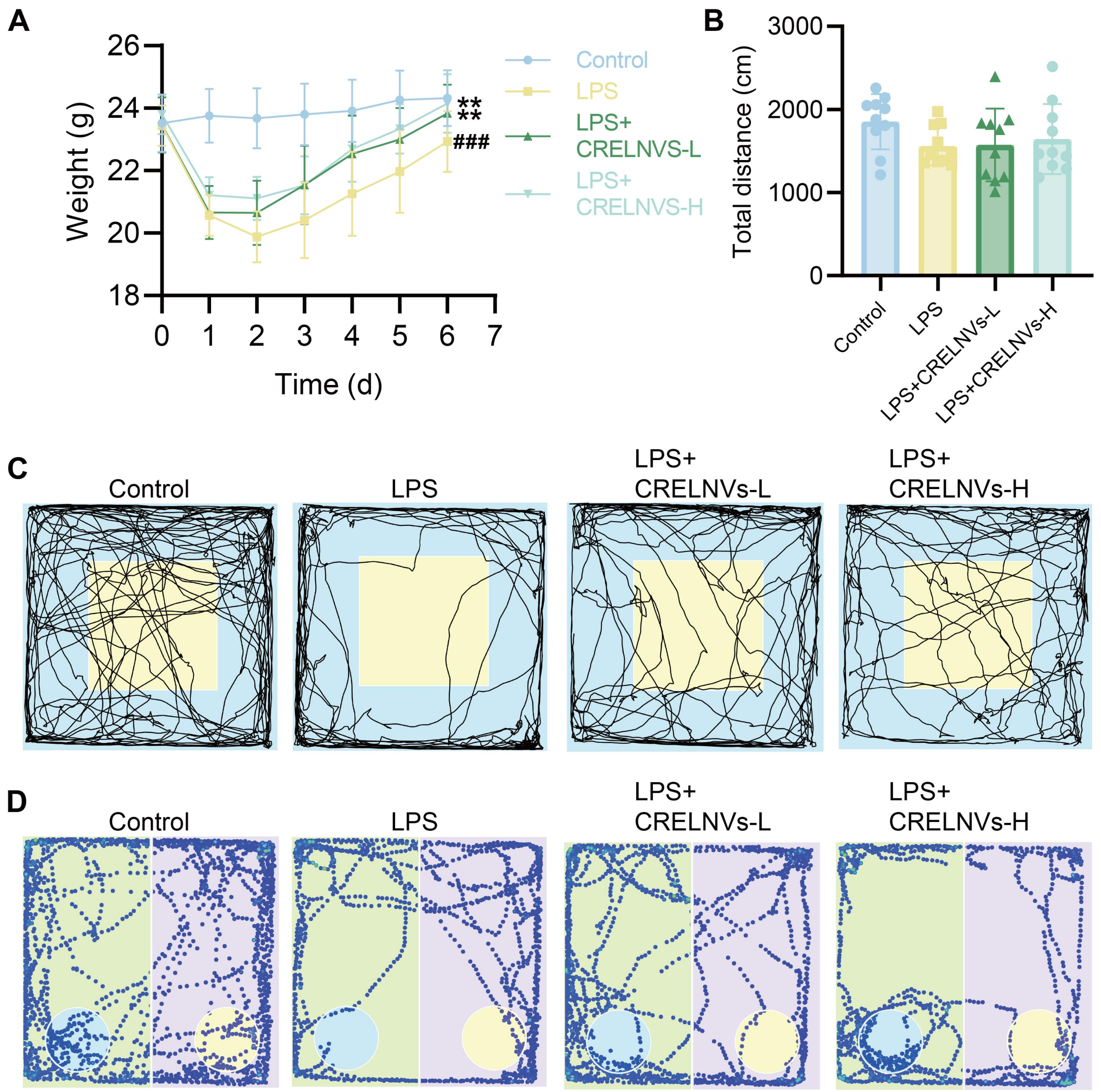

Figure S2 CRELNVs mitigated memory impairment in the hippocampus of SAE mice. A. Time course of body weight, $n = 10$. B. Total distances in open field test, $n = 10$. (C) the open field test and (D) novel-object recognition test movement traces. $^{\#}P < 0.05$, $^{\#\#}P < 0.01$, $^{\#\#\#}P < 0.001$ vs. control group; $^{*}P < 0.05$, $^{**}P < 0.01$, $^{***}P < 0.001$ vs. lipopolysaccharide (LPS) group

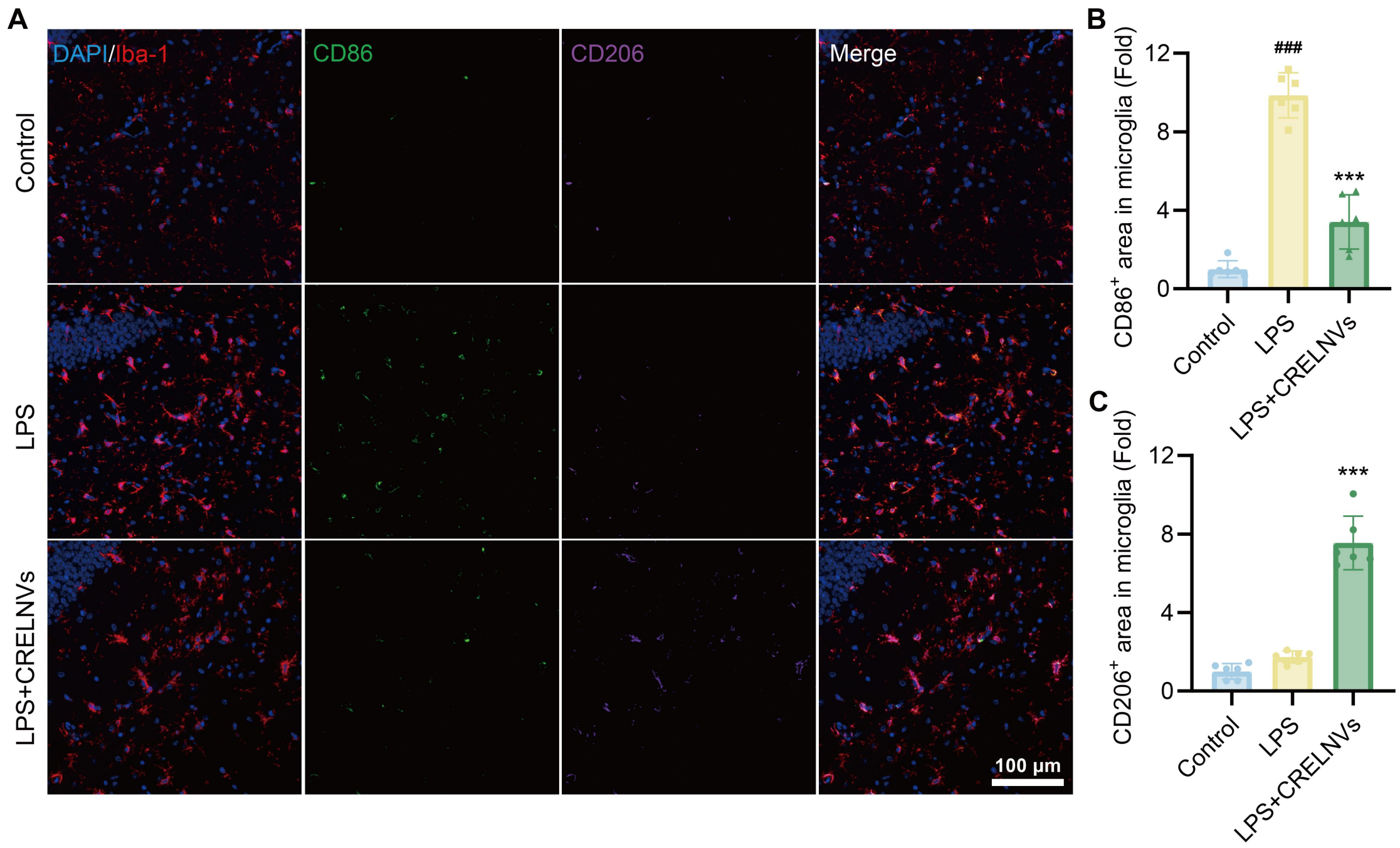

Figure S3 the microglial phenotype in the hippocampus of SAE mice. A. Representative images of immunofluorescence staining of Iba-1, CD86 and CD206 in the hippocampi of SAE mice, scale bar 100 μm. Quantification of the area of $CD86^{+}/Iba\text{-}1^{+}$ cells (B) and $CD206^{+}/Iba\text{-}1^{+}$ cells (C) in A, n = 6. $^{\#\#\#}P < 0.001$ *vs.* Control group; $^{***}P < 0.001$ *vs.* LPS group.

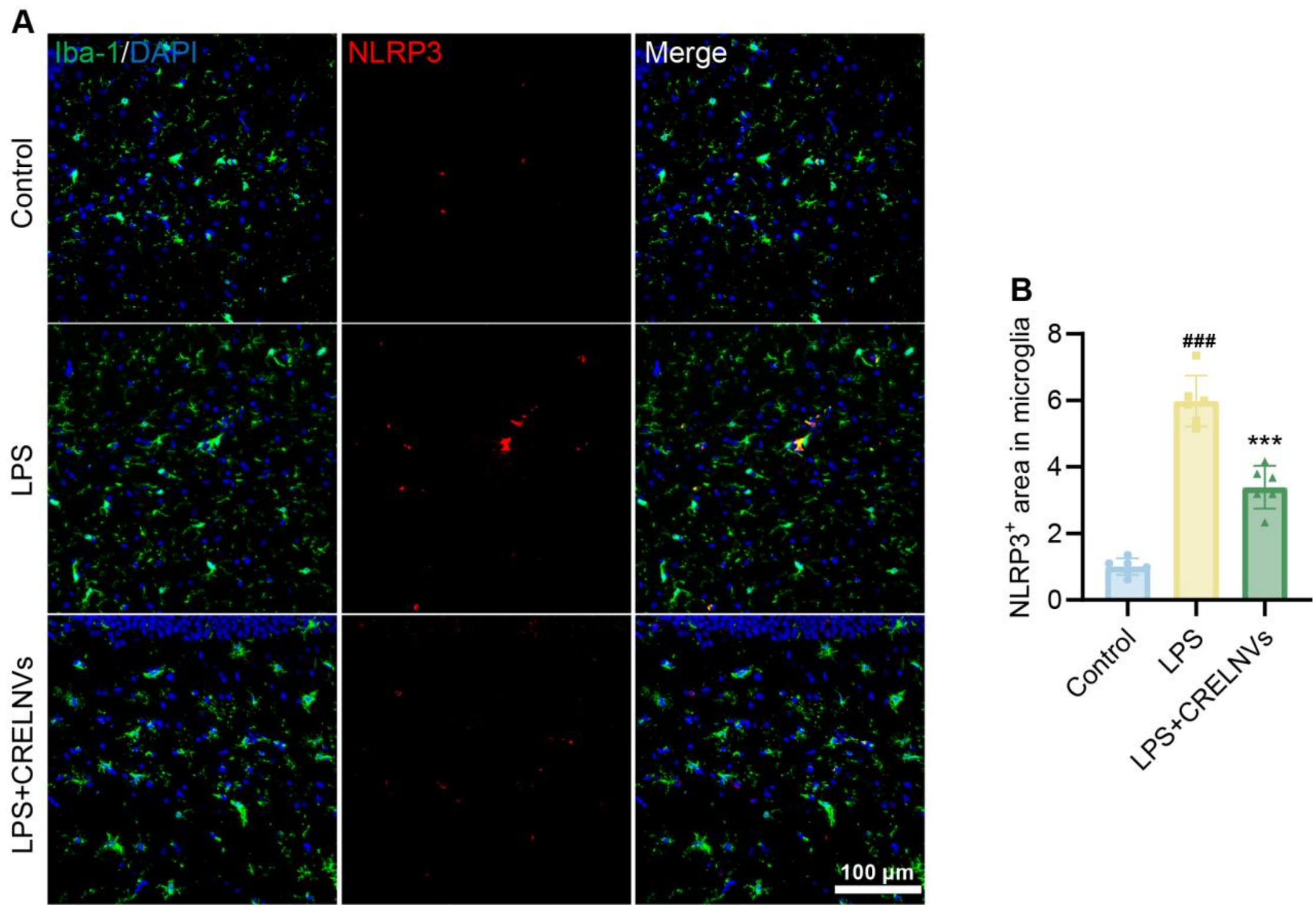

Figure S4 the NLRP3 expression in in the hippocampus of SAE mice. A. Representative images of immunofluorescence staining of Iba-1 and NLRP3 in the

hippocampi of SAE mice, scale bar 100 μm. B. Quantification of the area of NLRP3+/Iba-1+ cells, n=6. $^{###}P$ < 0.001 *vs.* Control group; ****P* < 0.001 *vs.* LPS group

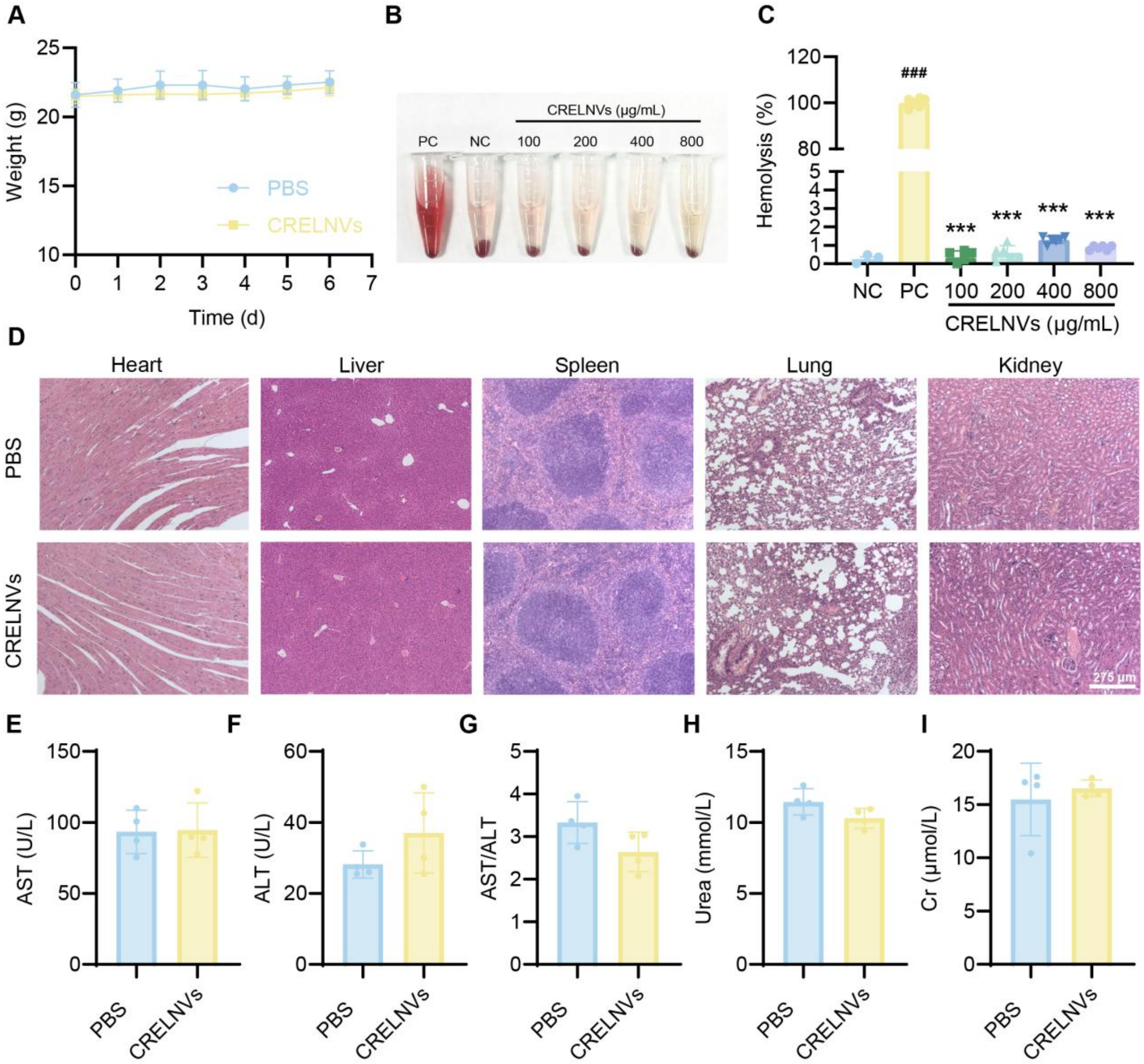

Figure S5 In vivo biosafety of CRELNVs. A. Body Weight, n=4. (B) Schematic diagram of the hemolysis test and (C) the Hemolysis rate, n=6; D. H&E staining, scale bar 275 μm. (E) AST, (F) ALT, (G) AST/ALT, (H) Urea and (I) Cr levels in the serum of SAE mice, n=4. $^{###}P$ < 0.001 *vs.* NC group; ****P* < 0.001 *vs.* PC group

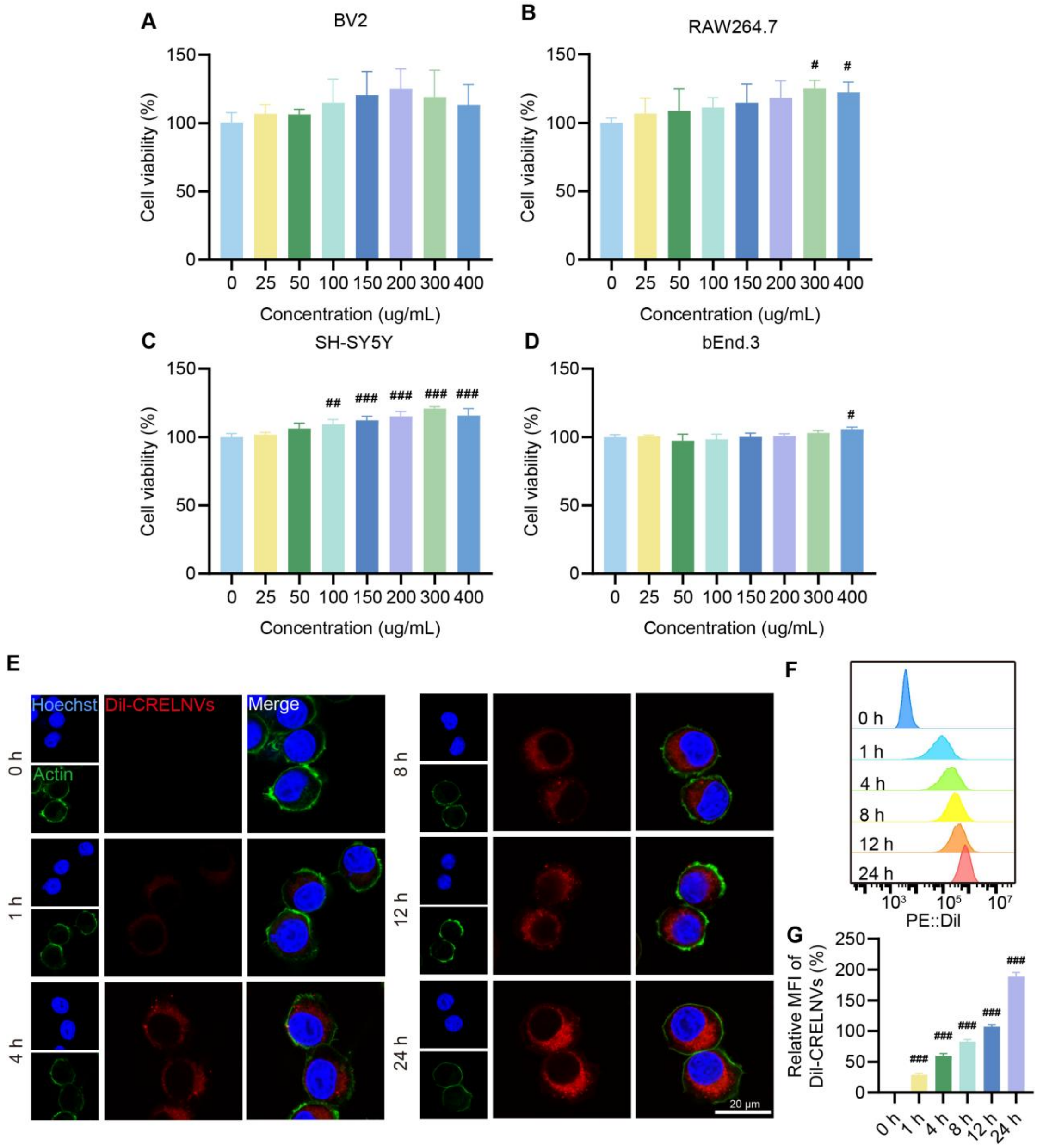

Figure S6. Cytotoxicity and cellular uptake of CRELNVs in vitro. CCK8 assay detecting cytotoxicity of CRELNVs on (A) BV2, (B) RAW264.7, (C)SH-SY5Y, and (D) bEnd.3 cells, n=4. E Representative confocal laser scanning microscopy images of Dil-CRELNVs uptake by BV2 cells, scale bar = 20 μm. (F)Flow cytometry peak plots and (G)quantification of Dil-CRELNVs uptake by BV2 cells, n=3. $^{\#}P < 0.05$, $^{\#\#}P < 0.01$, $^{\#\#\#}P < 0.001$ *vs.* Negtive Control group

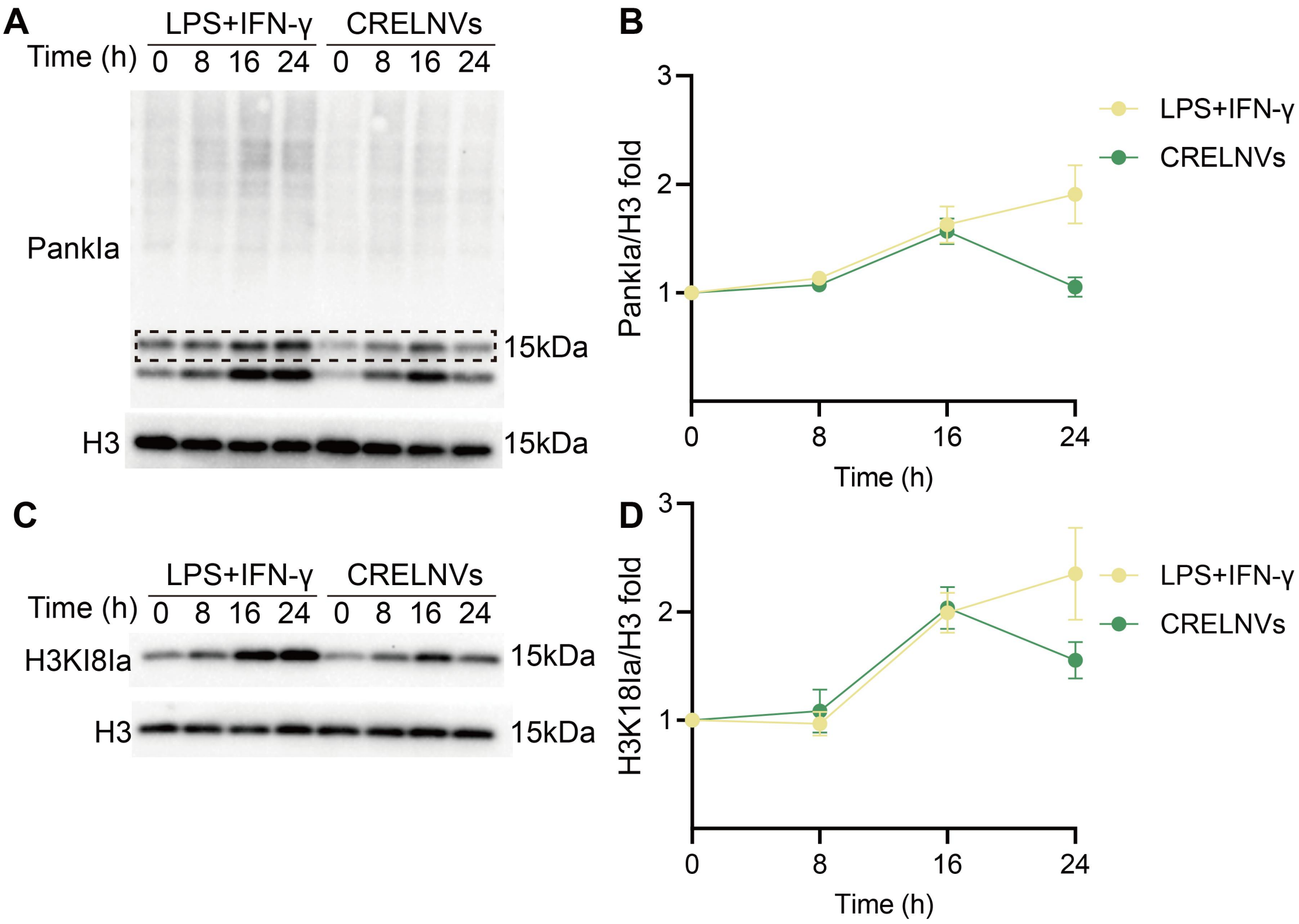

Figure S7. CRELNVs downregulated lactate levels and inhibited histone lactylation (H3K18la) in vitro. A, B. Western blot analysis: Pan-KIa and H3 levels in BV2 cells, n = 3. C, D. Western blot analysis: H3K18 and H3 levels in BV2 cells, n = 3. $^{\#}P$ < 0.05, $^{\#\#}P$ < 0.01, $^{\#\#\#}P$ < 0.001 vs. control group; *P < 0.05, **P < 0.01, ***P < 0.001 vs. LPS group.

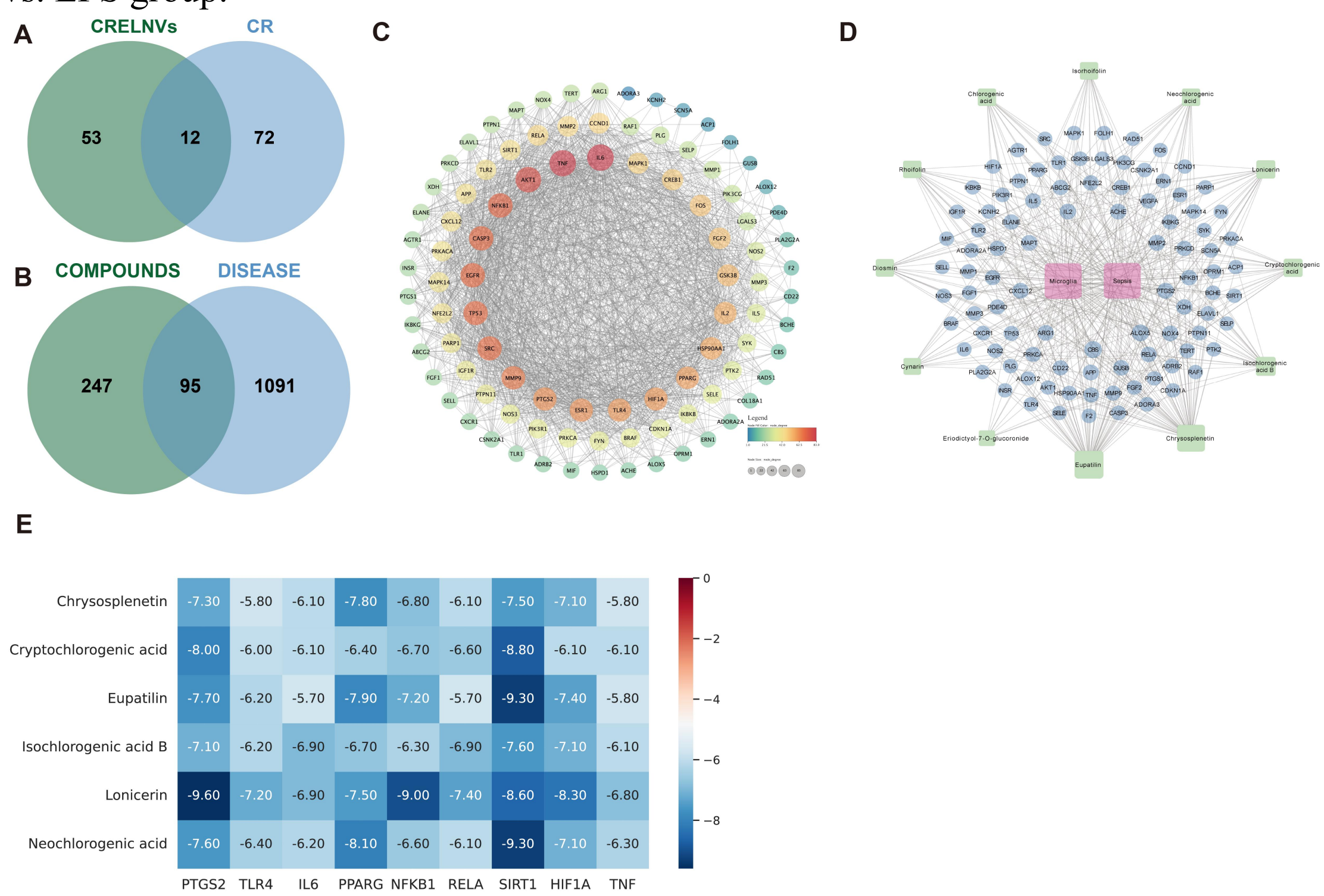

Figure S8. CRELNVs exert inflammatory response effects on SAE via NF-κB. A. Venn diagram of the 12 common compounds in CRELNVs and *C. morifolium*. B.

Venn diagram of the 95 targets in CRELNVs and sepsis. C. Antisepsis PPI network of CRELNVs. Larger areas indicate larger nodes, colors closer to red indicate stronger associations, lighter colors indicate weaker associations, and the core target is located in the inner circle. D. Herb-component-target network of panel C. E. Binding energy heatmap of molecular docking for 12 common compounds and the NF-κB-involved target.

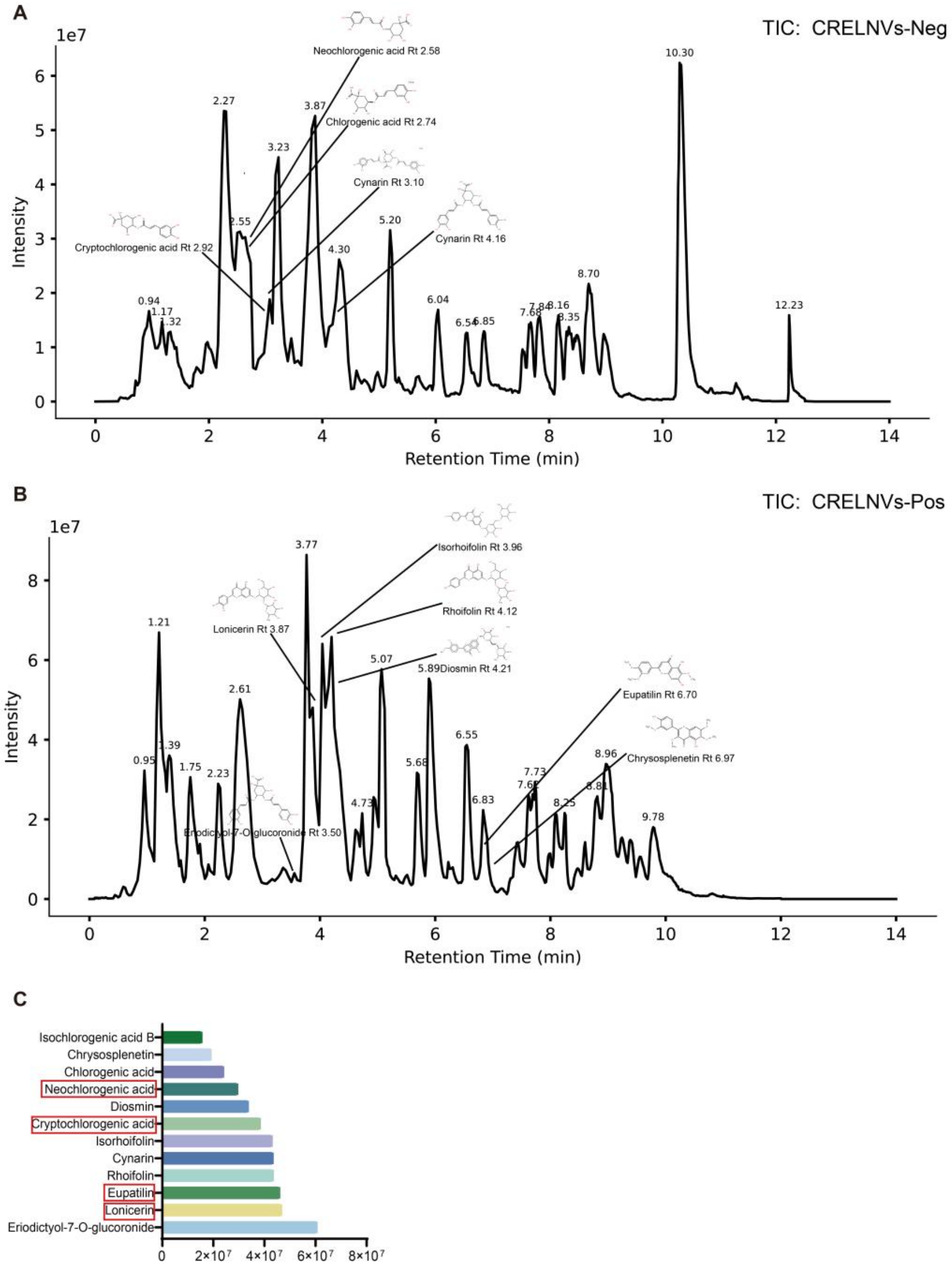

Figure S9. Proportions of various compounds in CRELNVs. Total ion flow diagram of CRELNVs with LC-MS/MS in (A) negative and (B) positive ion modes. C. Relative contents of 12 active compounds in CRELNVs.

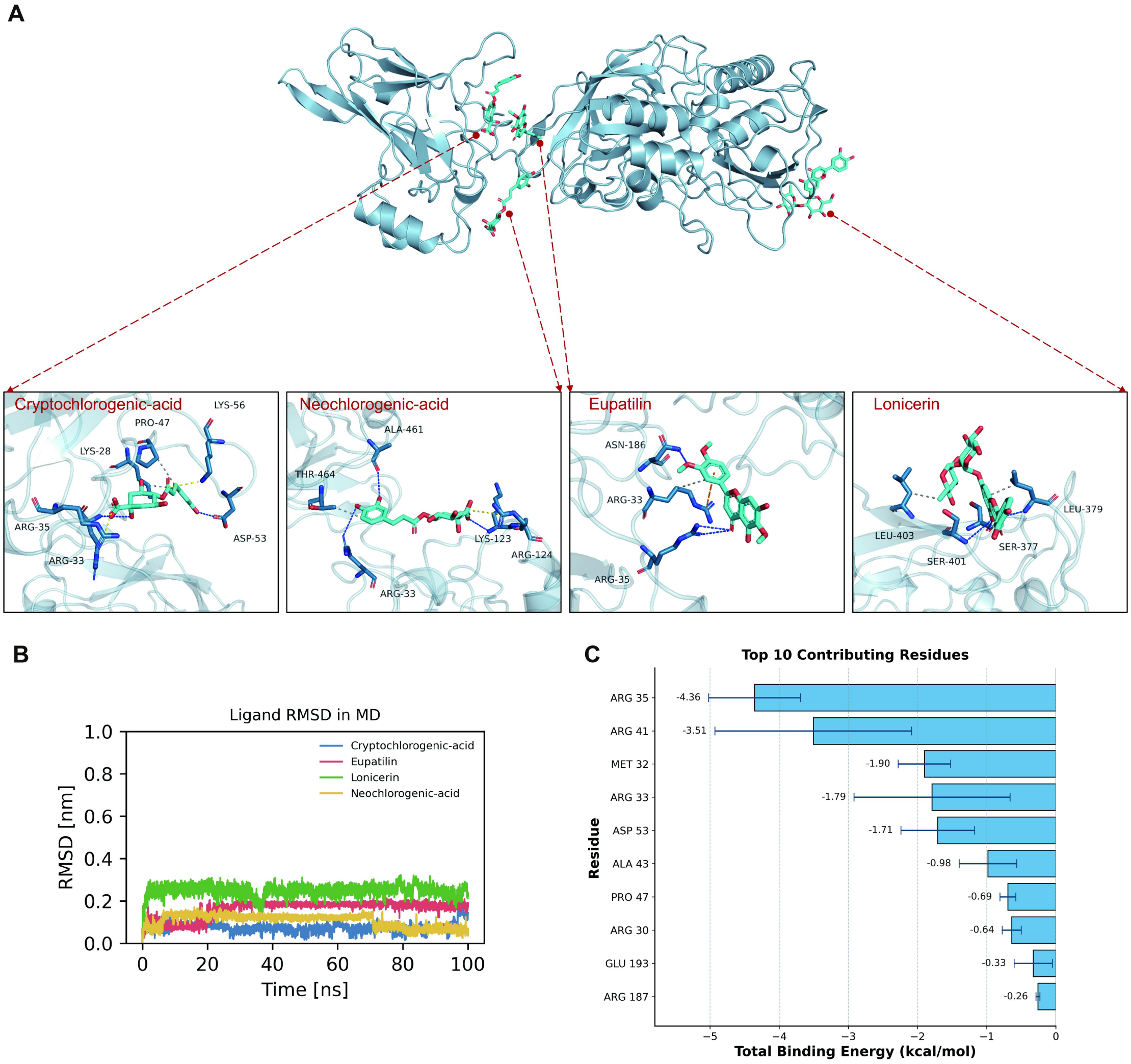

Figure S10. the binding region of complex. A. Best docking pockets in NF-κB by Boltz-2. B. Time course of the root mean square deviation of the ligand-NF-κB. C. Total contributing residues of the complex.
