# Supplementary material for: Chrysanthemum Morifolium–Derived Nanovesicles Reduce Lactate‐Associated Histone Lactylation and Modulate Microglia‐Mediated Neuroinflammation in Sepsis‐Associated Encephalopathy
Source: Adv Sci (Weinh). 2026 Oct 14:e78330. Online ahead of print. doi: 10.1002/advs.78330 (PMC13669446; doi:10.1002/advs.78330)
Supplement: Supplementary file 2 [file ADVS-9999-e78330-s003.docx]

Table S2 The visualizations of CRELNVs-compounds-target in top 6.

| group | 3D structure | Binding site | Docking Energy (kcal/mol) |
| --- | --- | --- | --- |
| PTGS2_Lonicerin |  |  | -9.20±0.35 |
| SIRT1_Eupatilin |  |  | -9.30±0.00 |
| SIRT1_Neochlorogenic acid |  |  | -8.73±0.21 |
| NFKB1_Lonicerin |  |  | -7.93±0.65 |
| SIRT1_Cryptochlorogenic acid |  |  | -7.23±0.21 |
| SIRT1_Lonicerin |  |  | -8.57±0.21 |

Table S3 the content of the 4 active compounds in CRELNVs (Lonicerin, Eupatilin, Cryptochlorogenic acid and Neochlorogenic acid)

| Name | CAS | Class Ⅰ | Sample Content (ug/mg) |
| --- | --- | --- | --- |
| Lonicerin | 25694-72-8 | Flavonoids | 8.42±0.09 |
| Eupatilin | 22368-21-4 | Flavonoids | 0.18±0.01 |
| Cryptochlorogenic-acid | 905-99-7 | Phenolic acids | 5.20±0.07 |
| Neochlorogenic-acid | 906-33-2 | Phenolic acids | 2.48±0.04 |

Table S4 the Binding free energies and energy components predicted by MM/GBSA in the complex of active components (kcal/mol).

| **System name** | Lonicerin | Eupatilin | Cryptochlorogenic-acid | Neochlorogenic-acid |
| --- | --- | --- | --- | --- |
| **Δ*E*_vdw_** | -31.27±1.64 | -29.59±2.31 | -23.97±4.28 | -29.45±3.66 |
| **Δ*E*_elec_** | -29.50±4.40 | -21.69±2.47 | -54.89±13.69 | -28.53±12.08 |
| **ΔG_GB_** | 38.95±4.46 | 38.58±3.70 | 55.71±8.57 | 35.42±7.16 |
| **ΔG_SA_** | -4.51±0.20 | -3.77±0.29 | -4.73±0.14 | -5.03±0.22 |
| **ΔG_bind_** | -26.35±2.69 | -16.48±1.10 | -27.89±3.37 | -27.59±2.01 |

ΔE_vdW_: van der Waals energy.

ΔE_elec_: electrostatic energy.

ΔG_GB_: electrostatic contribution to solvation.

ΔG_SA_: non-polar contribution to solvation.

ΔG_bind_: binding free energy.

Table S5 the Binding free energies and energy components predicted by MM/GBSA in the single active components (kcal/mol).

| **System name** | Lonicerin | Eupatilin | Cryptochlorogenic-acid | Neochlorogenic-acid |
| --- | --- | --- | --- | --- |
| **Δ*E*_vdw_** | -28.31± 4.22 | -27.77±2.58 | -18.71± 2.87 | -19.19±1.83 |
| **Δ*E*_elec_** | -48.91±10.91 | -11.22±5.52 | -100.20±12.67 | 83.84±3.33 |
| **ΔG_GB_** | 57.96± 7.23 | 25.11±7.30 | 101.11±10.70 | -72.99±3.14 |
| **ΔG_SA_** | -4.49± 0.29 | -3.18±0.40 | -3.67±0.11 | -2.84±0.18 |
| **ΔG_bind_** | -23.76± 4.03 | -17.07±1.98 | -21.47±2.58 | -11.19±1.45 |

ΔE_vdW_: van der Waals energy.

ΔE_elec_: electrostatic energy.

ΔG_GB_: electrostatic contribution to solvation.

ΔG_SA_: non-polar contribution to solvation.

ΔG_bind_: binding free energy.

Table S6 Primer sequences for qRT-PCR analysis

| **Gene** | **Primer (5’-3’)** |
| --- | --- |
| IL-1β-Forward | TGCCACCTTTTGACAGTGATG |
| IL-1β-Reverse | AAGGTCCACGGGAAAGACAC |
| IL-6-Forward | CCAAGAGGTGAGTGCTTCCC |
| IL-6-Reverse | CTGTTGTTCAGACTCTCTCCCT |
| TNF-α-Forward | ACCCTCACACTCACAAACCA |
| TNF-α-Reverse | ATAGCAAATCGGCTGACGGT |
| IL-10-Forward | GCTCTTACTGACTGGCATGAG |
| IL-10-Reverse | CGCAGCTCTAGGAGCATGTG |
| iNOS-Forward | GAGCTGGGCTGTACAAACCTT |
| iNOS-Reverse | CATTGGAAGTGAAGCGTTTCG |
| Arg-1-Forward | CTCCAAGCCAAAGTCCTTAGAG |
| Arg-1-Reverse | AGGAGCTGTCATTAGGGACATC |
| GAPDH- Forward | GGTCCCAGCTTAGGTTCATCA |
| GAPDH-Reverse | CCAATACGGCCAAATCCGTTC |
